# Supplementary material for: Does moderate alcohol consumption accelerate the progression of liver disease in NAFLD? A systematic review and narrative synthesis
Source: BMJ Open. 2022 Jan 4;12(1):e049767. doi: 10.1136/bmjopen-2021-049767 (PMC8728442; doi:10.1136/bmjopen-2021-049767)
Supplement: Supplementary data [file bmjopen-2021-049767supp001.pdf]

Medline(Ovid)

1. (((fatty or fat or steato*) adj3 (liver* or hepat*)) or steatohepat* or (visceral adj2 steato*)).ti,ab.
2. non-alcoholic fatty liver disease/
3. fatty liver/
4. (nafl* or nash).ti,ab.
5. non?alcoholic steato*.mp. [mp=title, abstract, original title, name of substance word, subject heading word, floating sub-heading word, keyword heading word, organism supplementary concept word, protocol supplementary concept word, rare disease supplementary concept word, unique identifier, synonyms]
6. (non?alcoholic adj3 (liver or fat*)).mp. [mp=title, abstract, original title, name of substance word, subject heading word, floating sub-heading word, keyword heading word, organism supplementary concept word, protocol supplementary concept word, rare disease supplementary concept word, unique identifier, synonyms]
7. or/1-6
8. exp Alcohol Drinking/
9. Alcoholism/ or Alcoholic Beverages/
10. (alcohol adj2 (unit* or consum* or level* or mg or g)).ti,ab.
11. (moderat* adj3 alcohol*).mp. [mp=title, abstract, original title, name of substance word, subject heading word, floating sub-heading word, keyword heading word, organism supplementary concept word, protocol supplementary concept word, rare disease supplementary concept word, unique identifier, synonyms]
12. or/8-11
13. 7 and 12
14. liver disease.mp. [mp=title, abstract, original title, name of substance word, subject heading word, floating sub-heading word, keyword heading word, organism supplementary concept word, protocol supplementary concept word, rare disease supplementary concept word, unique identifier, synonyms]
15. fibrosis.mp. [mp=title, abstract, original title, name of substance word, subject heading word, floating sub-heading word, keyword heading word, organism supplementary concept word, protocol supplementary concept word, rare disease supplementary concept word, unique identifier, synonyms]
16. (scar* adj3 liver).mp. [mp=title, abstract, original title, name of substance word, subject heading word, floating sub-heading word, keyword heading word, organism supplementary concept word, protocol supplementary concept word, rare disease supplementary concept word, unique identifier, synonyms]
17. cicatrix.mp. [mp=title, abstract, original title, name of substance word, subject heading word, floating sub-heading word, keyword heading word, organism supplementary concept word, protocol supplementary concept word, rare disease supplementary concept word, unique identifier, synonyms]
18. (end-stage adj3 liver).mp. [mp=title, abstract, original title, name of substance word, subject heading word, floating sub-heading word, keyword heading word, organism supplementary concept word, protocol supplementary concept word, rare disease supplementary concept word, unique identifier, synonyms]
19. ((liver or biliary) adj cirrhosis).mp. [mp=title, abstract, original title, name of substance word, subject heading word, floating sub-heading word, keyword heading word, organism supplementary concept word, protocol supplementary concept word, rare disease supplementary concept word, unique identifier, synonyms]
20. (hepatic insufficiency or liver failure or end stage liver disease or hepatic failure or hepatic encephalopathy or hepatic impairment).mp. [mp=title, abstract, original title, name of substance

word, subject heading word, floating sub-heading word, keyword heading word, organism supplementary concept word, protocol supplementary concept word, rare disease supplementary concept word, unique identifier, synonyms]
21. (mortality or death or dead or deceased or passed away).mp. [mp=title, abstract, original title, name of substance word, subject heading word, floating sub-heading word, keyword heading word, organism supplementary concept word, protocol supplementary concept word, rare disease supplementary concept word, unique identifier, synonyms]
22. ((hepatocellular or liver cell) adj carcinoma).mp. [mp=title, abstract, original title, name of substance word, subject heading word, floating sub-heading word, keyword heading word, organism supplementary concept word, protocol supplementary concept word, rare disease supplementary concept word, unique identifier, synonyms]
23. or/14-22
24. 13 and 23
25. limit 24 to yr="1990-Current"

Embase(Ovid)

1. (((fatty or fat or steato*) adj3 (liver* or hepat*)) or steatohepat* or (visceral adj2 steato*)).ti,ab.
2. exp nonalcoholic fatty liver/
3. (nafl* or nash).ti,ab.
4. non?alcoholic steato*.mp. [mp=title, abstract, heading word, drug trade name, original title, device manufacturer, drug manufacturer, device trade name, keyword, floating subheading word, candidate term word]
5. (non?alcoholic adj3 (liver or fat*)).mp. [mp=title, abstract, heading word, drug trade name, original title, device manufacturer, drug manufacturer, device trade name, keyword, floating subheading word, candidate term word]
6. or/1-5
7. drinking behavior/
8. exp alcoholism/
9. exp alcoholic beverage/
10. (alcohol adj2 (unit* or consum* or level* or mg or g)).ti,ab.
11. (moderat* adj3 alcohol*).mp. [mp=title, abstract, heading word, drug trade name, original title, device manufacturer, drug manufacturer, device trade name, keyword, floating subheading word, candidate term word]
12. or/7-11
13. 6 and 12
14. liver disease.mp. [mp=title, abstract, heading word, drug trade name, original title, device manufacturer, drug manufacturer, device trade name, keyword, floating subheading word, candidate term word]
15. fibrosis.mp. [mp=title, abstract, heading word, drug trade name, original title, device manufacturer, drug manufacturer, device trade name, keyword, floating subheading word, candidate term word]
16. (scar* adj3 liver).mp. [mp=title, abstract, heading word, drug trade name, original title, device manufacturer, drug manufacturer, device trade name, keyword, floating subheading word, candidate term word]
17. cicatrix.mp. [mp=title, abstract, heading word, drug trade name, original title, device manufacturer, drug manufacturer, device trade name, keyword, floating subheading word, candidate term word]

18. ((hepatcellular or liver cell) adj carcinoma).mp. [mp=title, abstract, heading word, drug trade name, original title, device manufacturer, drug manufacturer, device trade name, keyword, floating subheading word, candidate term word]
19. (end-stage adj3 liver).mp. [mp=title, abstract, heading word, drug trade name, original title, device manufacturer, drug manufacturer, device trade name, keyword, floating subheading word, candidate term word]
20. (Mortality or death or dead or deceased or passed away).mp. [mp=title, abstract, heading word, drug trade name, original title, device manufacturer, drug manufacturer, device trade name, keyword, floating subheading word, candidate term word]
21. ((liver or biliary) adj cirrhosis).mp. [mp=title, abstract, heading word, drug trade name, original title, device manufacturer, drug manufacturer, device trade name, keyword, floating subheading word, candidate term word]
22. (hepatic insufficiency or liver failure or end stage liver disease or hepatic failure or hepatic encephalopathy or hepatic impairment).mp. [mp=title, abstract, heading word, drug trade name, original title, device manufacturer, drug manufacturer, device trade name, keyword, floating subheading word, candidate term word]
23. or/14-22
24. 13 and 23
25.
26. limit 25 to yr="1990 -Current"

Cochrane library

ID Search Hits
#1 (((fatty or fat or steato*) NEAR/3 (liver* or hepat*)) or steatohepat* or (visceral NEAR/2 steato*))
#2 MeSH descriptor: [Non-alcoholic Fatty Liver Disease] this term only
#3 MeSH descriptor: [Fatty Liver] this term only
#4 (nafl* or nash)
#5 non?alcoholic steato*
#6 (non?alcoholic NEAR/3 (liver or fat*))
#7 #1 or #2 or #3 or #4 or #5 or #6
#8 MeSH descriptor: [Alcohol Drinking] explode all trees
#9 MeSH descriptor: [Alcoholism] this term only
#10 MeSH descriptor: [Alcoholic Beverages] this term only
#11 (alcohol NEAR/2 (unit* or consum* or level* or mg or g))
#12 (moderat* NEAR/3 alcohol)
#13 #8 or #9 or #10 or #11 or #12
#14 #7 AND #13
#15 Liver disease
#16 Fibrosis
#17 cicatrix or (scar* NEAR/3 liver)
#18 (end-stage NEAR/3 liver)
#19 ((Liver or biliary) NEAR cirrhosis)
#20 (hepatic insufficiency or liver failure or end stage liver disease or hepatic failure or hepatic encephalopathy or hepatic impairment)
#21 (Mortality or death or dead or deceased or passed away)
#22 ((hepatocellular or liver cell) NEAR carcinoma)
#23 #15 or #16 or #17 or #18 or #19 or #20 or #21 or #22

#24 #14 AND #23 with Cochrane Library publication date Between Jan 1990 and Dec 2019

CINAHL(EBSCO)

S1 TI ((((fatty or fat or steato*) N3 (liver* or hepat*)) or steatohepat* or (visceral N2 steato*))) OR AB ((((fatty or fat or steato*) N3 (liver* or hepat*)) or steatohepat* or (visceral N2 steato*))) OR (MH "Nonalcoholic Fatty Liver Disease") OR (MH "Fatty Liver") OR TI ((nafl* or nash)) OR AB ((nafl* or nash)) OR non#alcoholic steato* OR ((non-alcoholic N3 (liver or fat*)))
S2 (MH "Alcoholic Drinking+") OR (((MH "Alcoholism") or (MH "Alcoholic Beverages"))) OR TI ((alcohol N2 (unit* or consum* or level* or mg or g))) OR AB ((alcohol N2 (unit* or consum* or level* or mg or g))) OR AB (moderat* N3 alcohol)
S3 S1 AND S2
S4 (Liver Diseases OR Liver Failure OR ((Liver or biliary) N1 Cirrhosis) OR Fibrosis OR Cicatrix OR (scar* N3 liver) OR (end-stage N3 liver) OR hepatic insufficiency OR end stage liver disease OR hepatic failure OR hepatic encephalopathy OR hepatic impairment OR Mortality OR death OR dead OR deceased OR passed away OR ((Hepatocellular OR liver cell) N1 carcinoma))
S5 S3 AND S4

Web of Science (CPCI)

1. TS = (((fatty or fat or steato*_ NEAR/3 (liver* or hepat*)) or steatohepat* or (visceral NEAR/2 steato*))
2. TS = non-alcoholic fatty liver disease
3. TS = fatty liver
4. TS = (nafl* or nash)
5. TS = non-alochol steato*
6. TS = (non-alcoholic NEAR/3 (liver or fat*))
7. #1 OR #2 OR #3 OR #4 OR #5 OR #6
8. TS = alcohol drinking
9. TS = (alcoholism or alcoholic beverages)
10. TS = (alcohol NEAR/2 (unit* or consum* or level* or mg or g))
11. TS = (moderate NEAR/3 alcohol*)
12. #8 OR #9 OR #10 OR #11
13. #7 AND #12
14. TS = liver disease
15. TS = fibrosis
16. TS = cicatrix
17. TS = (end-stage NEAR/3 liver)
18. TS = liver cirrhosis
19. TS = hepatic insufficiency
20. #14 OR #15 OR #16 OR #17 OR #18 OR #19
21. #13 AND #20
