# Supplementary material for: Does moderate alcohol consumption accelerate the progression of liver disease in NAFLD? A systematic review and narrative synthesis
Source: BMJ Open. 2022 Jan 4;12(1):e049767. doi: 10.1136/bmjopen-2021-049767 (PMC8728442; doi:10.1136/bmjopen-2021-049767)
Supplement: Author's manuscript [file bmjopen-2021-049767.draft_revisions.pdf]

BMJ Open is committed to open peer review. As part of this commitment we make the peer review history of every article we publish publicly available.

When an article is published we post the peer reviewers' comments and the authors' responses online. We also post the versions of the paper that were used during peer review. These are the versions that the peer review comments apply to.

The versions of the paper that follow are the versions that were submitted during the peer review process. They are not the versions of record or the final published versions. They should not be cited or distributed as the published version of this manuscript.

BMJ Open is an open access journal and the full, final, typeset and author-corrected version of record of the manuscript is available on our site with no access controls, subscription charges or pay-per-view fees (http://bmjopen.bmj.com).

If you have any questions on BMJ Open's open peer review process please email info.bmjopen@bmj.com

BMJ Open

Does moderate alcohol consumption accelerate the progression of liver disease in NAFLD? A systematic review and narrative synthesis

Journal:	*BMJ Open*
Manuscript ID	bmjopen-2021-049767
Article Type:	Original research
Date Submitted by the Author:	08-Feb-2021

Complete List of Authors:	Jarvis, Helen; Newcastle University, Population Health Sciences Institute<br>O'Keefe, Hannah; Newcastle University, Population Health Sciences Institute<br>Craig, Dawn; Newcastle University, Population Health Sciences Institute<br>Stow, Daniel; Newcastle University, Population Health Sciences Institute<br>Hanratty, Barbara; Newcastle University, Population Health Sciences Institute<br>Anstee, Quentin; Newcastle University, Translational and Clinical Research Institute

Keywords:	Hepatology < INTERNAL MEDICINE, GENERAL MEDICINE (see Internal Medicine), PRIMARY CARE

SCHOLARONE™
Manuscripts

I, the Submitting Author has the right to grant and does grant on behalf of all authors of the Work (as defined in the below author licence), an exclusive licence and/or a non-exclusive licence for contributions from authors who are: i) UK Crown employees; ii) where BMJ has agreed a CC-BY licence shall apply, and/or iii) in accordance with the terms applicable for US Federal Government officers or employees acting as part of their official duties; on a worldwide, perpetual, irrevocable, royalty-free basis to BMJ Publishing Group Ltd ("BMJ") its licensees and where the relevant Journal is co-owned by BMJ to the co-owners of the Journal, to publish the Work in this journal and any other BMJ products and to exploit all rights, as set out in our licence.

The Submitting Author accepts and understands that any supply made under these terms is made by BMJ to the Submitting Author unless you are acting as an employee on behalf of your employer or a postgraduate student of an affiliated institution which is paying any applicable article publishing charge ("APC") for Open Access articles. Where the Submitting Author wishes to make the Work available on an Open Access basis (and intends to pay the relevant APC), the terms of reuse of such Open Access shall be governed by a Creative Commons licence – details of these licences and which Creative Commons licence will apply to this Work are set out in our licence referred to above.

Other than as permitted in any relevant BMJ Author's Self Archiving Policies, I confirm this Work has not been accepted for publication elsewhere, is not being considered for publication elsewhere and does not duplicate material already published. I confirm all authors consent to publication of this Work and authorise the granting of this licence.

For peer review only

Does moderate alcohol consumption accelerate the progression of liver disease in NAFLD? A systematic review and narrative synthesis

Helen Jarvis[1] (0000-0001-5039-0228) , Hannah O'Keefe[1] (0000-0002-0107-711X), Dawn Craig[1], Daniel Stow[1], *Barbara Hanratty[1], *Quentin M Anstee[2]

1. Population Health Sciences Institute, Faculty of Medical Sciences, Newcastle University, Newcastle upon Tyne, UK, NE2 4HH

Helen Jarvis helen.jarvis2@ncl.ac.uk

NIHR Doctoral Research Fellow

Hannah O'Keefe hannah.o'keefe@newcastle.ac.uk

Training Fellow in Evidence Synthesis

Dawn Craig (dawn.craig@newcastle.ac.uk)

Professor of Practice in Evidence Synthesis

Daniel Stow (daniel.stow@newcastle.ac.uk)

Research Associate

Barbara Hanratty (barbara.hanratty@newcastle.ac.uk)

Professor of Primary Care and Public Health

2. Translational and Clinical Research institute, Faculty of Medical Sciences, Newcastle University, Newcastle upon Tyne, UK, NE2 4HH

Quentin M Anstee (quentin.anstee@newcastle.ac.uk)

Professor of Experimental Hepatology

* Joint last authorship BH and QMA

1. Correspondence to: Helen Jarvis, GP and NIHR DRF, Population Health Sciences Institute, Faculty of Medical Sciences, Newcastle University, Newcastle upon Tyne, UK, NE2 4HH. Email: helen.jarvis2@ncl.ac.uk

For peer review only

Structured abstract:

Objectives:

Liver disease is a leading cause of premature death, partly driven by the increasing incidence of Non-alcohol related fatty liver disease (NAFLD). Many people with a diagnosis of NAFLD drink moderate amounts of alcohol. There is limited guidance for clinicians looking to advise these patients on the effect this will have on their liver disease progression. This review synthesises the evidence on moderate alcohol consumption and its potential to predict liver disease progression in people with diagnosed NAFLD.

Methods:

A systematic review of longitudinal observational cohort studies was conducted. Databases (MEDLINE, EMBASE, The Cochrane Library and ClinicalTrials.gov) were searched up to September 2020. Studies were included that reported progression of liver disease in adults with NAFLD, looking at moderate levels of alcohol consumption as the exposure of interest. Risk of bias was assessed using the QUIPS tool.

Results:

Of 4241 unique citations, 6 met the inclusion criteria. Pooling of data was not possible due to heterogeneity and studies were analysed using narrative synthesis. Evidence suggested that any level of alcohol consumption is associated with worsening of liver outcomes in NAFLD, even for drinking within recommended limits. Well conducted population based studies estimated up to a doubling of incident liver disease outcomes in NAFLD patients drinking at moderate levels.

Conclusions:

This review found that any level of alcohol intake in NAFLD may be harmful to liver health. Study heterogeneity in definitions of alcohol exposure as well as in outcomes limited quantitative pooling of results. Use of standardised definitions for exposure and outcomes would support future meta-analysis.

Based on this synthesis of the most up to date longitudinal evidence, clinicians seeing patients with NAFLD should currently advise abstinence from alcohol.

Funding and registration:

For peer review only

This study was funded by an NIHR DRF (NIHR300716). The protocol was registered with PROSPERO (#CRD42020168022).

Strengths and limitations of this study:

- Up to date synthesis of the best available evidence on the role of moderate alcohol consumption in NAFLD.
- Clear protocol limiting inclusion to longitudinal evidence to study temporal associations.
- Study heterogeneity in definitions of alcohol exposure and outcomes limited quantitative pooling of results.
- Only six studies met the inclusion criteria; further studies using standardized definitions are needed in this area to support meta-analysis

Main Text:

Introduction:

Liver disease is an increasing health burden across the world, and it is now a major cause of premature (<65) mortality (1,2). As premature mortality rates from many non-communicable diseases have fallen over the last 30 years, the burden of liver disease is increasing (2,3). The commonest causes of chronic liver disease in high income countries are alcohol related liver disease (ARLD) and metabolic-syndrome related liver disease (or non-alcohol related fatty liver disease - NAFLD). Whilst the labelling of liver disease suggests a dichotomy, the clinical reality is that there is significant overlap between ARLD and NAFLD (4). The incidence of obesity and diabetes is rising, and a substantial proportion of the population is drinking alcohol at above recommended limits (5). It is estimated that up to 17% of the adult population may meet criteria for both NAFLD and ARLD (6). Despite this, there is little guidance available for generalist healthcare professionals, on how to advise people with a diagnosis of NAFLD on safer alcohol consumption.

Recommendations on safe alcohol consumption levels vary worldwide. Increasingly, they take into account the effect that alcohol has on the risk of developing many adverse health outcomes, including cancer. International analysis suggests this should be as low as total

For peer review only

abstinence to minimize all health risks (7). Recommended limits for safe alcohol consumption in the UK general population are up to 14 units of alcohol per week in both males and females (8), which equates to 16 grams of alcohol per day at 8g/unit. Moderate alcohol consumption is generally defined in the literature as drinking within, or slightly in excess of, these limits versus complete abstinence (4). There is a significant gap between this recommended 'moderate' limit and the levels of alcohol consumption that would prompt an assessment for alcohol-related liver damage. The UK National Institute of Health and Care Excellence (NICE) recommends offering a liver cirrhosis test to men drinking over 50 units and women drinking over 35 units a week on an ongoing basis over several months (9), leaving a significant proportion who are drinking at and above 14 units a week, but below the levels to have liver assessment based on their alcohol consumption alone.

There is still uncertainty, and an absence of guidance, on safe levels of alcohol consumption for people with established NAFLD. Indeed, it is not clear that any level of alcohol consumption is safe to minimise progression of the liver disease in this population. It is known that people with very high levels of alcohol consumption (who would meet criteria for a diagnosis of ARLD), and who also have metabolic risk factors, are at even greater risk of adverse liver outcomes (10,11). But there is also some evidence that for people with metabolic risk factors (but who do not have a NAFLD diagnosis), drinking alcohol at low levels may protect against cardiovascular disease, prevent fatty liver disease, and lead to better outcomes than with complete abstinence (12,13).

The purpose of this systematic review is to synthesise evidence on the role of moderate alcohol consumption on progression to severe liver disease in people with diagnosed NAFLD. This will help guide the advice given to NAFLD populations around safe alcohol consumption in primary care and specialist settings.

Methods:

The protocol for this review was registered in advance with PROSPERO (International Prospective Register of Systematic Reviews, #CRD42020168022).

Types of studies, inclusion and exclusion criteria

For peer review only

Primary studies were included if they were prospective or retrospective cohort studies. The population of interest was adult patients (>18 years old) with diagnosed NAFLD. The outcome of interest was progression of liver disease in this population. The exposure of interest was no vs moderate alcohol consumption. For our inclusion criteria we defined ‘moderate consumption’ as up to 35 units per week in females, and 50 units per week in males (levels that would be considered the threshold for definite risk of ARLD according to NICE guidelines (9)). This definition included studies that focused on the effects of alcohol within or just above current weekly recommended limits (the usual definition of ‘moderate’ alcohol consumption), as well as those who looked beyond these levels of consumption, up to the NICE ARLD levels.

Exclusion criteria were as follows: 1) studies where the population had diagnosed ARLD; 2) studies where the population was defined according to their alcohol consumption levels rather than their NAFLD status at baseline; 3) studies where patients already had severe liver disease at the time of cohort entry; 4) cross sectional studies or studies where exposure was only measured at the same time as outcome.

We performed a systematic review following the Preferred Reporting Items for Systematic Reviews and Meta-Analyses (PRISMA) guidelines (14).

Search strategy and data extraction

Potentially relevant studies were identified through systematic literature searches of relevant databases (MEDLINE, EMBASE, The Cochrane Library and ClinicalTrials.gov, CPCI-S (Conference Proceedings Citation Index – Science, Web of Knowledge), OpenGrey (http://www.opengrey.eu/)) in January 2020 and updated in September 2020. No language restrictions were applied, and databases were searched from 1990 onwards. Reference lists from potentially relevant papers and previous review articles were hand searched. MeSH (Medical Subject Headings) and free text terms for the NAFLD population, alcohol exposures and liver outcomes of interest were used. Two researchers (HJ and either HO or DS) independently screened titles and abstracts. Any disagreement in full text selection was resolved by consensus. Record screening was also assisted by Rayyan, an online software tool that assesses similarities between selected records and highlights other potentially relevant studies based on the screener’s previous selection (15). Full texts of potentially relevant papers were obtained and read by two independent researchers with reference to the predefined set of

For peer review only

criteria to identify final study inclusion. Data were extracted into a standardised form, piloted on 3 studies before full extraction. Data extraction was based on the updated checklist for critical appraisal and data extraction for systematic reviews of prediction studies (CHARMS-PF) checklist for prognostic studies (16), undertaken by one researcher and checked by a second. Two authors (HJ, HO) assessed the risk of bias independently. Since the included studies were observational cohort studies of prognostic factors, the QUIPS (Quality in Prognostic factor Studies) tool was used (17).

Data synthesis

Pooling of data was not possible due to heterogeneity across studies. A narrative synthesis (18) was undertaken, with data synthesised by alcohol exposure level. Due to the small number of studies, even those with high risk of bias are included in the synthesis, although this bias assessment is made clear throughout the narrative.

Patient and Public Involvement

Patients and the Public were not involved in the the design or conduct of this review but will be involved in the dissemination of findings through a funded PPI steering group and close collaboration with the British Liver Trust.

Results:

The searches identified 4241 unique citations. Of the titles and abstracts screened, 34 articles were selected for full-text screening. Twenty-eight were excluded for reasons reported in the PRISMA diagram (Figure 1). A total of six unique studies representing data from five cohorts were eligible for inclusion in the systematic review, and were assessed for quality (Figure 1), (19-24).

Characteristics of included studies

Further details of included studies are shown in Table 1.

For peer review only

Table 1: Characteristics of included studies

Author /year	Country	Study design and population	Yrs f/u	Method of NAFLD diag*	Method of measuring alcohol consumption	Definition of moderate consumption studied as RF	Study outcomes of interest and event no	Adjustments of interest considered	Adjusted HRs/OR for liver events with 95% CI and *p*-values	Risk of bias
Aberg 2019 (19)	Finland	retrospective data linkage cohort analysis NAFLD population 6462, mean age 53 yrs, 60% M	10.9	FLI >30	Questionnaire at cohort entry	< 50g/day in 10 g categories with abstinence as reference	composite non-fatal and fatal liver disease 58 events	? unclear other than age, sex	per increase in 10g of alcohol per day from abstinence HR 1.43 (1.12 - 1.82) p0.004	high
Aberg 2020 (20)	Finland (FINRISK Health survey)	retrospective data linkage cohort analysis NAFLD population 8345, mean age 53.7 yrs, 60% M	11.1	FLI >60	Questionnaire at cohort entry (recall for past month)	< 50g/day in 10 g categories with abstinence as reference	composite non-fatal and fatal liver disease 152 events	age, sex, smoking, T2DM	g alcohol/day v abstinence<br>0-9 HR 1.38 (0.74-2.58)<br>10-19 HR 2.18(1.04-4.53)<br>20-29 HR 3.62(1.67-7.76)<br>30-39 HR 3.53(1.53-8.14)<br>40-49 HR8.79(3.95-19.56)	low
Ajmera 2018 (21)	USA	retrospective analysis of longitudinal cohorts within NASH CRN NAFLD population 285, mean age 47 yrs, 30% M	3.9	liver biopsy	Questionnaire at cohort entry (Skinner lifetime drinking history)	<2 drinks per day and excluded if >6 drinks on 1 occasion >/= monthly	histological progress on follow up biopsy	age, sex, race, smoking	OR of outcome persistent moderate drinkers v abstinence**<br>resolution of NASH OR 0.32 (0.11-0.92) p0.04<br>fibrosis progression OR 0.00 (-0.29-0.29) p0.99	mod
Chang 2019 (22)	South Korea (Kangbuk Samsung Health Study)	prospective population cohort NAFLD population 58927, mean age 37.7, 82% M	4.9	US	Questionnaire at each study visit (annual or biennial)	10 - 19.9 g/day (F)<br>10 - 29.9 g/day (M)<br>(low 1-9.9g/day)	fibrosis progress as estimated by high indirect serum scores***	age, sex, BMI, smoking, exercise level, education, T2DM, BP	mod v abstinence$ (repeat observations)<br>Fib4: HR 1.33(1.13-1.57)<br>NFS: HR 1.37(1.23-1.52)<br>low v abstinence (repeat observations)<br>Fib 4: HR 1.08(0.91-1.27)	low

For peer review only - http://bmjopen.bmj.com/site/about/guidelines.xhtml

									NFS: HR 1.14(1.02-1.27)	
Ekstedt 2009 (23)	Sweden	retrospective cohort NAFLD population 71, mean age 47.3, 72% M	13.8	US and liver biopsy	Questionnaire AUDIT-C and interview at follow up	g/day - no upper limit defined as 'moderate'	fibrosis progress on follow up biopsy	age, sex, BMI, T2DM, fibrosis at baseline	increasing alcohol g/week OR 1.012 (1.000-1.025) p0.055	low
Kawamura 2016 (24)	Japan	prospective cohort NAFLD population 9959, mean age 49, 87% M (included 18 patients >70g alcohol/day defined as ARLD)	5.4	US	Questionnaire at baseline and every 6 months	g/day in categories with <20g/day as reference	HCC on imaging	age, sex, BMI, T2DM, serum markers	g/day alcohol v <20g/day 20-39 HR 0.90(0.11-7.90) p0.919 ≥40-69 HR 2.48(1.01-6.05) p0.047 >70 HR 12.61(5.68-28.00) p0.001	low

Abbreviations: NAFLD = non-alcohol related fatty liver disease; NASH = non-alcohol related steatohepatitis; ARLD = alcohol related liver disease; RF= risk factor; CRN= clinical research network; Yrs = years; M = Male; FLI = Fatty Liver Index; US = hepatic ultrasound; g = grams; HCC = hepatocellular carcinoma; BMI = body mass index; T2DM = type 2 diabetes mellitus; BP = blood pressure; HR = Hazard ratio; OR = Odds ratio; CI = confidence interval; NFS = NAFLD fibrosis score.

**Note multiple differences in means and OR presented for different histological and biochemical outcomes between abstainers, persistent moderate drinkers, and changes in alcohol consumption between biopsies. Presented data represent histological outcomes of potential clinical prognostic significance within the remit of this review.

*** Scores used to estimate fibrosis progression were the Fib4 score, NAFLD fibrosis score (NFS) and APRI score
$ multiple HR presented in paper for different score outcomes for single and repeated outcome measures looking at intermediate/high- or high-risk scores in low and moderate drinkers and different subgroups. Presented data represent outcomes best in keeping with remit of this review using widely used indirect serum markers of liver fibrosis.

For peer review only

Within the studies meeting inclusion criteria, three (21,22,23) looked at the exposure of alcohol consumption up to, or similar to, the accepted international definition of ‘moderate consumption’. This is <20g/day in women and <30g/day in men (25). Three of the studies (19,20,24) looked at low alcohol consumption but also extended ‘moderate consumption’ up to levels of alcohol consumption which would be considered more consistent with ARLD. Table 2 shows international definitions of moderate alcohol consumption, UK recommended limits and levels that would warrant assessment for ARLD, all expressed in grams of alcohol and UK units.

Table 2: alcohol consumption definitions and equivalences

**Definitions:**	grams of alcohol		UK Units of alcohol	
	Daily*	Weekly*	Daily*	Weekly*
Accepted International consensus of moderate alcohol consumption	**F: <20**	F: <140	F: < 2.5	F: <17.5
	**M: <30**	M: <210	M: < 3.75	M: < 26.25
UK recommended safe weekly limits	</= 16	</= 112	</= 2	**</= 14**
NICE thresholds for assessing for liver cirrhosis	F: > 40	F :> 280	F: > 5	**F: > 35**
	M:> 57	M: >400	M: > 7.1	**M: > 50**

* Daily and weekly figures are given for comparison only. The bold numbering for each definition is the standard format in which this definition is expressed

Moderate alcohol consumption (accepted international definitions) and risk of liver disease progression in NAFLD

Three studies examined the effects of alcohol in NAFLD using definitions in keeping with the accepted international definition of ‘moderate consumption’ (21,22,23). Although these studies shared a similar aim, they varied in NAFLD population definition, measurement of alcohol consumption and choice of liver outcomes. Two looked at histological progression outcomes and one used non-invasive indirect blood-based markers of liver fibrosis. Two of the studies

For peer review only

were rated as having a low risk of bias (22,23) and one was rated as having a moderate risk (21).

Ajmera et al (21) studied a NAFLD population taken retrospectively from the NASH clinical research network, including populations from an observational study and the placebo arm of two NASH drug trials, all of whom had biopsy proven NAFLD (285 participants). Alcohol consumption was measured at cohort entry and at varying time points up to, and including, follow-up liver biopsy, which occurred, on average, four years later. Multiple histological markers of disease progression were studied, and the authors looked at the association between baseline drinking status and disease progression as well as change in drinking status over time and disease progression. For most of the histological end points studied, there was no significant difference between moderate drinkers and abstainers in outcomes, with the only significant results suggesting that abstainers had less progressive or a higher likelihood of regression of their disease between biopsies, particularly the persistent abstainers. Results should be interpreted in the knowledge that a large number of related histological outcomes were reported, increasing the likelihood of a statistically significant result by chance. The absence of detailed information on which other prognostic factors were taken into account, led to a rating of moderate on risk of bias assessment.

A similar study by Ekstedt et al (23) looked at a smaller group (71 participants) of biopsy proven NAFLD, with follow up histology an average of 13.8 years after initial biopsy. Alcohol consumption was assessed at baseline and follow up, with heavy episodic drinking assessed in addition to weekly consumption. Primary outcome was significant fibrosis progression, defined as progression by one or more fibrosis stage or the development of end stage liver disease during follow up. Although higher weekly alcohol consumption showed some tendency to predict fibrosis progression (OR for increase in grams of alcohol per week 1.012 (1.000 - 1.025)) only the presence of heavy episodic drinking (defined as >60g/day in men and >48g/day in women more than once a month) reached statistical significance in predicting fibrosis progression.

Of note in both the Ajmera and Ekstedt studies were the very low levels of alcohol consumption in the ‘moderate drinkers’, with the majority (78%) of the moderate drinkers drinking less than monthly in the Ajmera study and the average weekly alcohol consumption in the Ekstedt study being only 39 g/week. Both studies also included a significant number of patients who already had liver inflammation (NASH) at baseline (over 50% in both studies), indicating a higher

For peer review only

proportion of patients with a tendency to progressive disease as compared to a general NAFLD population, as would be expected with biopsy-based studies.

In contrast to the relatively selective biopsy studies, Chang et al (22) studied a large prospective population cohort (Kangbuk Samsung Health Study) of whom 58,927 had ultrasound evidence of fatty liver but without evidence of other liver diagnoses or advanced disease. Alcohol exposure was weekly units at baseline and follow up was for a median of 8.3 years with outcome of interest being progression to advanced liver fibrosis using non-invasive blood-based markers of disease. For moderate drinkers (10 - 30 g/day), the risk of progressing to advanced fibrosis (using intermediate/high Fib4 score as the outcome) was HR 1.33 (1.13 - 1.57), when compared to abstainers. Light drinkers (1-10 g/day) showed a tendency towards more advanced disease when compared to abstainers, but this did not reach statistical significance (HR 1.08 CI 0.91-1.27).

Moderate alcohol consumption (below the threshold that would be consistent with ARLD) and risk of liver disease progression in NAFLD

Three studies extended the definition of moderate alcohol consumption beyond the international consensus definition of ‘moderate consumption’. Two of the studies were rated as having a low risk of bias (20,24), with one rated as high risk of bias (19).

The general population longitudinal data presented by Chang et al is supplemented by two recent related studies by Aberg et al (19,20), using data from the same Finnish National Health Surveys (FINRISK, Health 2000) cohort. The definition of moderate alcohol consumption was increased to include anything up to 50 grams per day in these studies. Although the exposures and outcome measures were the same in the two related studies, the NAFLD population was defined using different fatty liver index (FLI) cut offs values, generating overlapping but distinct study populations. For this reason, data are presented from both studies.

The first study, only available as a conference abstract, (19) used a FLI>30 to retrospectively define their NAFLD population. This low FLI would generally be used as a ‘rule out’ rather than ‘rule in’ cutoff for NAFLD diagnosis (26) and the limited data presented suggests that using abstinence as a reference, any increase in alcohol consumption by 10 g/day, increased incident liver events (combined fatal and non-fatal outcomes) by 43% with a presented HR of 1.43 (1.12 - 1.82) for each 10 g rise in daily alcohol consumption. The data presented contained few details

For peer review only

of adjustment factors or analysis plan. This study was graded as having a high risk of bias, and these results should be interpreted with caution.

A larger study (20), based on the same cohort, retrospectively identified a NAFLD population based on a FLI of >60 (the accepted and validated cut off for making a positive diagnosis of NAFLD in the literature (26)). Alcohol intake at cohort entry was based on estimated consumption over the previous year. Lifetime abstainers were used as the reference group. Fatal and non-fatal liver outcomes were studied in 8345 participants over 92 350 person years of follow up. The study concluded that incident liver disease is higher at all levels of alcohol consumption, compared to lifetime abstainers with steadily rising hazard ratios as the level of alcohol consumption increases. Although drinking up to 10g per day was not statistically significantly different to abstaining (HR 1.38 CI 0.74 -2.58 in the final model), levels of alcohol consumption between 10 g and 19 g, which are roughly equivalent to the 14 units per week recommended limits, prognosticated for over double the number of incident liver events in NAFLD patients (HR 2.18 CI 1.05-4.53). At higher levels, which would not necessarily trigger a liver assessment for alcohol related harm in current guidelines, risk of significant liver disease was nearly nine times higher (for consumption of 40 - 49 g of alcohol a day, HR 8.79 CI 3.95-19.56).

A retrospective Japanese cohort study also looked at stepwise rises in daily alcohol consumption as a prognostic factor for the more specific outcome of hepatocellular carcinoma (HCC) in people with fatty liver (identified on ultrasound). The Kawamura study (24) with 9959 participants followed for a median of nearly 2000 days, had a reference group of people drinking < 20g of alcohol per day, rather than abstainers. This differed from all the other studies reviewed. Only those drinking at between 40-69 g of alcohol a day had a statistically significant increase in rates of HCC (HR 2.48 CI 1.01 - 6.05 p 0.047), with no effect in those drinking at more moderate levels. The population in this retrospective cohort were patients undergoing ultrasound at two tertiary hepatology centres in Japan rather than a general population cohort, and as HCC is known to occur in non-cirrhotic NAFLD (27) comparison with outcomes from other studies should be interpreted with caution.

Excluding the only study rated as having a high risk of bias (19), the other good quality longitudinal studies of varying design, all reported either no association or a negative impact of moderate amounts of alcohol on future liver disease outcomes. This was seen across the

For peer review only

studies looking at levels of alcohol consumption within the international definition of moderate consumption, and those that extended this definition of moderate consumption.

Discussion:

Summary of results

In this systematic review of the latest available longitudinal data, we found evidence to suggest that any amount of alcohol, even at low levels, may be harmful for liver health in people with diagnosed NAFLD. This evidence comes from both general population-based cohorts using coded liver outcomes, as well as tertiary centre NAFLD populations defined using histological end points.

Comparison with existing literature

Until recently the majority of evidence in this area has come from cross-sectional studies where alcohol exposure was assessed at the same time as liver outcomes. These data provides somewhat contradictory results, with several studies indicating that moderate alcohol consumption is associated with lower levels of liver disease progression (28,29,30,31) although more recent studies support of our findings, and suggest the opposite (32,33). The design employed in these studies does not allow the assessment of temporal relationships and is open to reverse causality (those with liver damage may be newly abstaining from alcohol for example) in addition to recall and other biases. On the basis of these limitations, cross-sectional studies were excluded from this current review, although they have been widely cited in previous critical reviews in this area, before more recent longitudinal data were available.

In the historical absence of large prospective cohort studies and the impossibility of conducting a controlled trial in the area, comparative work has been undertaken using Mendelian randomisation. This utilises random genetic variations which affects the rate of alcohol metabolism as a proxy measure for alcohol exposure, with randomisation of NAFLD patients based on an allele known to confer lower lifetime alcohol consumption by necessity due to the unpleasant effects of drinking even low levels of alcohol. Findings from this study were supportive of our review, with the group with higher lifetime alcohol consumption showing

For peer review only

markers of more severe disease on biopsy, even though alcohol consumption was at very modest levels (34).

In addition to the evidence on the relationship between modest alcohol consumption in NAFLD and liver outcomes, other published studies have focused on overall mortality and cardiovascular outcomes. A study of 4264 participants in an ultrasound diagnosed NAFLD cohort study showed no significant difference in overall mortality in those with alcohol consumption in the low/moderate range versus abstinence after 20 years of follow up (35). A subsequent study with the same US cohort reported a protective effect of low alcohol consumption on overall survival in NAFLD (36). The evidence for a protective effect of low alcohol consumption on cardiovascular outcomes in the general population is generally accepted (37). The evidence for cardiovascular protection in those with NAFLD is more limited, with some evidence that moderate alcohol may provide some benefit (38) but more recent studies finding no protective effects (32,39). The comparative evidence on overall mortality and cardiovascular outcomes highlights the need to assess liver disease risks within these competing contexts.

Strengths and limitations

Although there have been several recent critical reviews of the role of moderate alcohol consumption in NAFLD, the most recent of which reach similar conclusions (4,40,41), these have been wider in their remit with less well defined inclusion criteria and less systematic methodology. The predetermined inclusion criteria, robust systematic data collection and reporting techniques (in line with PRISMA guidelines) and decision to avoid cross-sectional data are all important in providing the best available evidence to answer the review question of the temporal relationship between moderate alcohol consumption and liver outcomes in NAFLD. The challenges of synthesising observational data, including unmeasured confounding and heterogeneity, were anticipated, but meant that data pooling was not possible.

A particular limitation hindering comparison between studies were the methods of defining moderate alcohol consumption. The consensus for defining a level of alcohol consumption above which a diagnosis of pure NAFLD cannot be made have been supported by the European Association for the Study of the Liver (EASL) and the American Association for the Study of the Liver Diseases (AASLD) and set at 20g/day in women and 30g/day in men (25,42),

For peer review only

yet most of the published studies do not use these cut-offs in their data. Until this is standardised across studies, with an additional consensus defining levels above this 'moderate' but not high enough to reach levels associated with a definite diagnosis of ARLD, synthesising the evidence in this area will remain challenging.

Implications for research/practice

This review adds weight to individual studies showing that any level of alcohol intake in NAFLD may be harmful to liver health. Further prospective cohort studies are needed, with detailed definitions/measures of alcohol exposure, and validated clinical liver outcomes, measured at appropriate times. Future research should focus on looking at outcomes in relation to accepted alcohol intake levels used in definitions of NAFLD. It should also take into account that the clinical reality is a dual-aetiology patient who may currently be excluded from both diagnostic categories based on their alcohol intake being too high for NAFLD, and too low for ARLD definitions. This is an ever-expanding patient group seen in many clinical settings.

Based on the evidence synthesised in this review, clinicians seeing patients with NAFLD in primary or secondary care should currently advise abstinence from alcohol to avoid accelerating liver harm. This is likely to be difficult for patients to accept, and public health messaging will need careful thought if it is to have any impact on liver health.

Author contribution statement:

HJ: conceptualization, methodology, conduct, analysis, writing of initial draft
HO: methodology, conduct, figures/infographic, critical review and comments on drafts
DC: supervision of methodology, conduct, critical review and comments on drafts
DS: methodology, conduct, critical review and comments on drafts
BH: conceptualization, supervision of methodology, conduct, analysis, critical review and comments on drafts
QA: conceptualization, supervision of conduct and analysis, critical review and comments on drafts

Competing interest statement:

All authors have completed the Unified Competing Interest form and declare that the study was funded by an NIHR Doctoral Research Fellowship (HJ personal award). There were no financial

For peer review only

relationships with any organisations that might have an interest in the submitted work in the previous three years. All authors report no other relationships or activities that could appear to have influenced the submitted work.

HJ reports grants from National Institute for Health research (NIHR), during the conduct of the study; personal fees from Intercept Pharma, personal fees from Norgine , outside the submitted work;
QMA reports grants from European Commission, during the conduct of the study; other from Acuitas Medical, grants, personal fees and other from Allergan/Tobira, other from E3Bio, other from Eli Lilly & Company Ltd, other from Galmed, grants, personal fees and other from Genfit SA, personal fees and other from Gilead, other from Grunthal, other from Imperial Innovations, grants and other from Intercept Pharma Europe Ltd, other from Inventiva, other from Janssen, personal fees from Kenes, other from MedImmune, other from NewGene, grants and other from Pfizer Ltd, other from Raptor Pharma, grants from GlaxoSmithKline, grants and other from Novartis Pharma AG, grants from Abbvie, personal fees from BMS, grants from GSK, other from NGMBio, other from Madrigal, other from Servier, outside the submitted work;

Funding statement:

This work was supported by and NIHR Doctoral Research Fellowship – HJ personal award. Award no: NIHR300716.

Data Sharing statement:

All data relevant to the study are included in the article or uploaded as supplementary information

References:

1. Williams R, Aspinall R, Bellis M, et al. Addressing liver disease in the UK: a blueprint for attaining excellence in health care and reducing premature mortality from lifestyle issues of excess consumption of alcohol, obesity, and viral hepatitis. Lancet. 2014;384(9958):1953–97.
2. GBD 2017 Cirrhosis Collaborators. The global, regional, and national burden of cirrhosis by cause in 195 countries and territories, 1990-2017: a systematic analysis for the Global Burden of Disease Study 2017. Lancet Gastroenterol Hepatol. 2020 Mar;5(3):245-266.

For peer review only

3. Martinez R, Lloyd-Sherlock P, Soliz P, et al. Trends in premature avertable mortality from non-communicable diseases for 195 countries and territories, 1990-2017: a population-based study. Lancet Glob Health 2020;8:e511-e523.
4. Boyle M, Masson S, Anstee QM. The bidirectional impacts of alcohol consumption and the metabolic syndrome: Cofactors for progressive fatty liver disease. J Hepatol. 2018;68(2):251–67.
5. Williams R, Alexander G, Armstrong I, et al. Disease burden and costs from excess alcohol consumption, obesity, and viral hepatitis: fourth report of the Lancet Standing Commission on Liver Disease in the UK. Lancet. 2017 Nov 28;
6. Baumeister SE, Alte D, Meyer C, John U. Health Risk drinking and problematic consumption of alcohol in Pomerania: comparative analysis of the Study of Health in Pomerania (SHIP) compared with the Federal German Health and Examination Survey in 1998. Gesundheitswesen. 2005 Jan;67(1):39–47.
7. Collaborators GBDA. Alcohol use and burden for 195 countries and territories, 1990-2016: a systematic analysis for the Global Burden of Disease Study 2016. Lancet 2018;392:1015-1035.
8. UK Chief Medical Officers' Low Risk Drinking Guidelines. Available at: https://assets.publishing.service.gov.uk/government/uploads/system/uploads/attachment_data/file/545937/UK_CMOs__report.pdf
9. Cirrhosis in over 16s: assessment and management | Guidance and guidelines | NICE. Available from: https://www.nice.org.uk/guidance/ng50
10. Hart CL, Morrison DS, Batty GD, Mitchell RJ, Davey Smith G. Effect of body mass index and alcohol consumption on liver disease: analysis of data from two prospective cohort studies. BMJ. 2010 Mar 11;340:c1240.
11. Liu B, Balkwill A, Reeves G, Beral V, Million Women Study Collaborators. Body mass index and risk of liver cirrhosis in middle aged UK women: prospective study. BMJ. 2010 Mar 11;340:c912.
12. Thun MJ, Peto R, Lopez AD, et al. Alcohol consumption and mortality among middle-aged and elderly U.S. adults. N Engl J Med. 1997 Dec 11;337(24):1705–14.
13. Sookoian S, Castaño GO, Pirola CJ. Modest alcohol consumption decreases the risk of non-alcoholic fatty liver disease: a meta-analysis of 43 175 individuals. Gut. 2014 Mar;63(3):530–2.

14. Moher D, Liberati A, Tetzlaff J, Altman DG, The PRISMA Group (2009). *Preferred Reporting Items for Systematic Reviews and Meta-Analyses:* The PRISMA Statement. PLoS Med 6(7): e1000097. doi:10.1371/journal.pmed1000097
15. Ouzzani M, Hammady H, Fedorowicz Z, Elmagarmid A. Rayyan-a web and mobile app for systematic reviews. Syst Rev. 2016 05;5(1):210.
16. Moons KGM, Groot JAH de, Bouwmeester W, et al. Critical Appraisal and Data Extraction for Systematic Reviews of Prediction Modelling Studies: The CHARMS Checklist. PLOS Medicine. 2014 Oct 14;11(10):e1001744.
17. Hayden JA, van der Windt DA, Cartwright JL, Côté P, Bombardier C. Assessing bias in studies of prognostic factors. Ann Intern Med. 2013 Feb 19;158(4):280–6.
18. Popay J, Roberts H, Sowden A, et al. Guidance on the conduct of narrative synthesis in systematic reviews: a product from the ESRC Methods Programme. Lancaster: Lancaster University. 2006;10(2.1):1018–4643
19. Åberg F, Puukka P, Sahlman P et al. In NAFLD, alcohol drinking habits and genetics predict progression to advanced liver disease: follow-up of population surveys. Journal of Hepatology. 2019; 70: e141. (poster abstract)
20. Åberg F, Puukka P, Salomaa V, et al. Risks of Light and Moderate Alcohol Use in Fatty Liver Disease: Follow-Up of Population Cohorts. Hepatology. 2020 Mar;71(3):835–48.
21. Ajmera V, Belt P, Wilson LA, et al. Among Patients With Nonalcoholic Fatty Liver Disease, Modest Alcohol Use Is Associated With Less Improvement in Histologic Steatosis and Steatohepatitis. Clin Gastroenterol Hepatol. 2018;16(9):1511-1520.e5.
22. Chang Y, Cho YK, Kim Y, et al. Nonheavy Drinking and Worsening of Noninvasive Fibrosis Markers in Nonalcoholic Fatty Liver Disease: A Cohort Study. Hepatology. 2019;69(1):64–75.
23. Ekstedt M, Franzén LE, Holmqvist M, et al. Alcohol consumption is associated with progression of hepatic fibrosis in non-alcoholic fatty liver disease. Scand J Gastroenterol. 2009;44(3):366–74.
24. Kawamura Y, Arase Y, Ikeda K, et al. Effects of Alcohol Consumption on Hepatocarcinogenesis in Japanese Patients With Fatty Liver Disease. Clin Gastroenterol Hepatol. 2016 Apr;14(4):597–605.
25. European Association for the Study of the Liver (EASL), European Association for the Study of Diabetes (EASD), European Association for the Study of Obesity (EASO). EASL-EASD-EASO Clinical Practice Guidelines for the Management of Non-Alcoholic Fatty Liver Disease. Obes Facts. 2016;9(2):65–90.

26. Bedogni G, Bellentani S, Miglioli L, et al. The Fatty Liver Index: a simple and accurate predictor of hepatic steatosis in the general population. BMC Gastroenterol. 2006 Nov 2;6:33.
27. Negro F. Natural history of NASH and HCC. Liver Int. 2020;40 Suppl 1:72–6.
28. Dunn W, Sanyal AJ, Brunt EM, et al. Modest alcohol consumption is associated with decreased prevalence of steatohepatitis in patients with non-alcoholic fatty liver disease (NAFLD). J Hepatol. 2012 Aug;57(2):384–91.
29. Hagström H, Nasr P, Ekstedt M, et al. Low to moderate lifetime alcohol consumption is associated with less advanced stages of fibrosis in non-alcoholic fatty liver disease. Scand J Gastroenterol. 2017 Feb;52(2):159–65.
30. Kwon HK, Greenson JK, Conjeevaram HS. Effect of lifetime alcohol consumption on the histological severity of non-alcoholic fatty liver disease. Liver Int. 2014 Jan;34(1):129–35.
31. Mitchell T, Jeffrey GP, MacQuillan GC, et al. The impact of alcohol consumption on hepatic fibrosis in non-alcoholic fatty liver disease. Hepatology. 2016. Vol 64 (supp) 548A (poster abstract)
32. Kashiwagi K, Yamaguchi A, Shiba S, et al. Moderate alcohol consumption is not associated with subclinical cardiovascular damage but with hepatic fibrosis in non-alcoholic fatty liver disease. Alcohol. 2020 Jul 30;89:1–7.
33. Mulazzani L, Alvisi M, Tovoli F, et al. Moderate alcohol consumption is associated with risk of fibrosis in patients with non-alcoholic fatty liver disease.Digestive and Liver Disease. 2020; 52 (0): pp e43-e44.
34. Sookoian S, Flichman D, Castaño GO, Pirola CJ. Mendelian randomisation suggests no beneficial effect of moderate alcohol consumption on the severity of nonalcoholic fatty liver disease. Aliment Pharmacol Ther. 2016;44(11–12):1224–34.
35. Younossi ZM, Stepanova M, Ong J, et al. Effects of Alcohol Consumption and Metabolic Syndrome on Mortality in Patients With Nonalcoholic and Alcohol-Related Fatty Liver Disease. Clin Gastroenterol Hepatol. 2018 Nov 23;
36. Hajifathalian K, Torabi Sagvand B, McCullough AJ. Effect of Alcohol Consumption on Survival in Nonalcoholic Fatty Liver Disease: A National Prospective Cohort Study. Hepatology. 2019;70(2):511–21.
37. Klatsky AL. Alcohol and cardiovascular diseases. Expert Review of Cardiovascular Therapy [Internet]. 2009 May 1 [cited 2020 Sep 14];7(5):499–506.

38. Sinn DH, Gwak G-Y, Cho J, et al. Modest alcohol consumption and carotid plaques or carotid artery stenosis in men with non-alcoholic fatty liver disease. Atherosclerosis. 2014 Jun 1;234(2):270–5.
39. VanWagner LB, Ning H, Allen NB, et al. Alcohol Use and Cardiovascular Disease Risk in Patients With Nonalcoholic Fatty Liver Disease. Gastroenterology. 2017;153(5):1260-1272.e3.
40. Ajmera VH, Terrault NA, Harrison SA. Is moderate alcohol use in nonalcoholic fatty liver disease good or bad? A critical review. Hepatology. 2017 Jun;65(6):2090–9.
41. Åberg F, Färkkilä M, Männistö V. Interaction Between Alcohol Use and Metabolic Risk Factors for Liver Disease: A Critical Review of Epidemiological Studies. Alcohol Clin Exp Res. 2020;44(2):384–403.
42. Chalasani N, Younossi Z, Lavine JE, et al. The diagnosis and management of non-alcoholic fatty liver disease: practice guideline by the American Gastroenterological Association, American Association for the Study of Liver Diseases, and American College of Gastroenterology. Gastroenterology. 2012 Jun;142(7):1592–609.

Figure 1: PRISMA diagram of study selection

For peer review only

Fig 1: PRISMA diagram of study selection

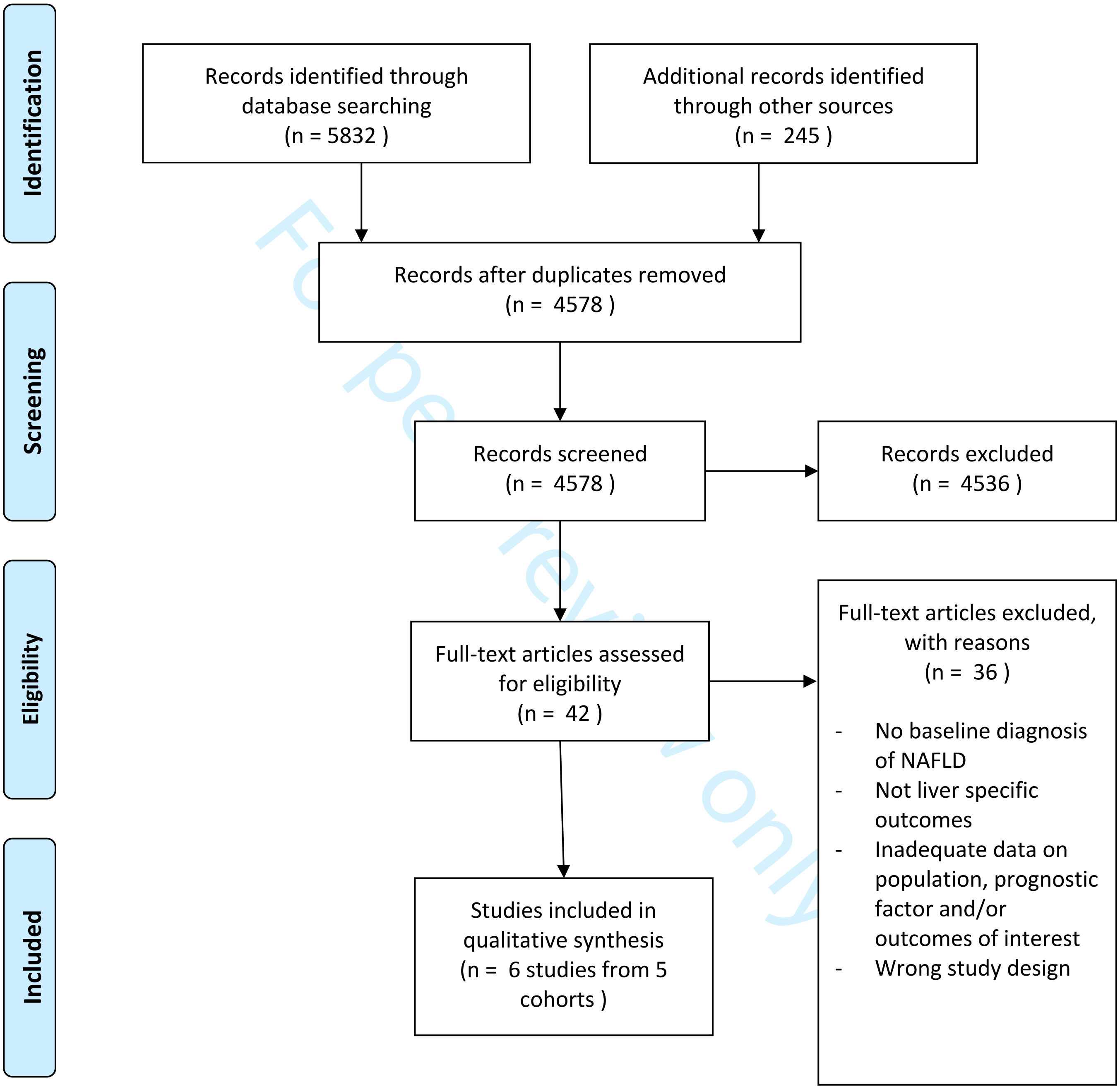

PRISMA 2009 Checklist

Section/topic	#	Checklist item	Reported on page #
**TITLE**			
Title	1	Identify the report as a systematic review, meta-analysis, or both.	Within title
**ABSTRACT**			
Structured summary	2	Provide a structured summary including, as applicable: background; objectives; data sources; study eligibility criteria, participants, and interventions; study appraisal and synthesis methods; results; limitations; conclusions and implications of key findings; systematic review registration number.	Abstract section para 1-4
**INTRODUCTION**			
Rationale	3	Describe the rationale for the review in the context of what is already known.	Introduction Para 1-3
Objectives	4	Provide an explicit statement of questions being addressed with reference to participants, interventions, comparisons, outcomes, and study design (PICOS).	Introduction Para 4
**METHODS**			
Protocol and registration	5	Indicate if a review protocol exists, if and where it can be accessed (e.g., Web address), and, if available, provide registration information including registration number.	Methods Para 1
Eligibility criteria	6	Specify study characteristics (e.g., PICOS, length of follow-up) and report characteristics (e.g., years considered, language, publication status) used as criteria for eligibility, giving rationale.	Methods – para 2-3
Information sources	7	Describe all information sources (e.g., databases with dates of coverage, contact with study authors to identify additional studies) in the search and date last searched.	Methods Para 3-4
Search	8	Present full electronic search strategy for at least one database, including any limits used, such that it could be repeated.	On request
Study selection	9	State the process for selecting studies (i.e., screening, eligibility, included in systematic review, and, if applicable, included in the meta-analysis).	Methods Para 2 Results: Fig 1
Data collection process	10	Describe method of data extraction from reports (e.g., piloted forms, independently, in duplicate) and any processes for obtaining and confirming data from investigators.	Methods Para 3,4
Data items	11	List and define all variables for which data were sought (e.g., PICOS, funding sources) and any assumptions and simplifications made.	Methods Para 2-3

For peer review only - http://bmjopen.bmj.com/site/about/guidelines.xhtml

PRISMA 2009 Checklist

Risk of bias in individual studies	12	Describe methods used for assessing risk of bias of individual studies (including specification of whether this was done at the study or outcome level), and how this information is to be used in any data synthesis.	Methods para 4-5
Summary measures	13	State the principal summary measures (e.g., risk ratio, difference in means).	N/A
Synthesis of results	14	Describe the methods of handling data and combining results of studies, if done, including measures of consistency (e.g., I^2) for each meta-analysis.	N/A

Page 1 of 2

Section/topic	#	Checklist item	Reported on page #
Risk of bias across studies	15	Specify any assessment of risk of bias that may affect the cumulative evidence (e.g., publication bias, selective reporting within studies).	N/A
Additional analyses	16	Describe methods of additional analyses (e.g., sensitivity or subgroup analyses, meta-regression), if done, indicating which were pre-specified.	N/A
**RESULTS**			
Study selection	17	Give numbers of studies screened, assessed for eligibility, and included in the review, with reasons for exclusions at each stage, ideally with a flow diagram.	Results Fig 1
Study characteristics	18	For each study, present characteristics for which data were extracted (e.g., study size, PICOS, follow-up period) and provide the citations.	Results Table 1
Risk of bias within studies	19	Present data on risk of bias of each study and, if available, any outcome level assessment (see item 12).	Results Table 1
Results of individual studies	20	For all outcomes considered (benefits or harms), present, for each study: (a) simple summary data for each intervention group (b) effect estimates and confidence intervals, ideally with a forest plot.	a) Table 1<br>b) N/A
Synthesis of results	21	Present results of each meta-analysis done, including confidence intervals and measures of consistency.	N/A
Risk of bias across studies	22	Present results of any assessment of risk of bias across studies (see Item 15).	N/A
Additional analysis	23	Give results of additional analyses, if done (e.g., sensitivity or subgroup analyses, meta-regression [see Item 16]).	N/A
**DISCUSSION**			
Summary of evidence	24	Summarize the main findings including the strength of evidence for each main outcome; consider their relevance to key groups (e.g., healthcare providers, users, and policy makers).	Discussion Para 1-3
Limitations	25	Discuss limitations at study and outcome level (e.g., risk of bias), and at review-level (e.g., incomplete retrieval of identified research, reporting bias).	Discussion para 5-6
Conclusions	26	Provide a general interpretation of the results in the context of other evidence, and implications for future research.	Discussion para 2,3,4 7,8

For peer review only - http://bmjopen.bmj.com/site/about/guidelines.xhtml

PRISMA 2009 Checklist

**FUNDING**			
Funding	27	Describe sources of funding for the systematic review and other support (e.g., supply of data); role of funders for the systematic review.	Title page

From: Moher D, Liberati A, Tetzlaff J, Altman DG, The PRISMA Group (2009). Preferred Reporting Items for Systematic Reviews and Meta-Analyses: The PRISMA Statement. PLoS Med 6(7): e1000097. doi:10.1371/journal.pmed1000097

For more information, visit: **www.prisma-statement.org**.

For peer review only

For peer review only - http://bmjopen.bmj.com/site/about/guidelines.xhtml

BMJ Open

Does moderate alcohol consumption accelerate the progression of liver disease in NAFLD? A systematic review and narrative synthesis

Journal:	*BMJ Open*
Manuscript ID	bmjopen-2021-049767.R1
Article Type:	Original research
Date Submitted by the Author:	27-Oct-2021
Complete List of Authors:	Jarvis, Helen; Newcastle University, Population Health Sciences Institute<br>O'Keefe, Hannah; Newcastle University, Population Health Sciences Institute<br>Craig, Dawn; Newcastle University, Population Health Sciences Institute<br>Stow, Daniel; Newcastle University, Population Health Sciences Institute<br>Hanratty, Barbara; Newcastle University, Population Health Sciences Institute<br>Anstee, Quentin; Newcastle University, Translational and Clinical Research Institute
**Primary Subject Heading**:	Gastroenterology and hepatology
Secondary Subject Heading:	Epidemiology, General practice / Family practice
Keywords:	Hepatology < INTERNAL MEDICINE, GENERAL MEDICINE (see Internal Medicine), PRIMARY CARE

SCHOLARONE™
Manuscripts

I, the Submitting Author has the right to grant and does grant on behalf of all authors of the Work (as defined in the below author licence), an exclusive licence and/or a non-exclusive licence for contributions from authors who are: i) UK Crown employees; ii) where BMJ has agreed a CC-BY licence shall apply, and/or iii) in accordance with the terms applicable for US Federal Government officers or employees acting as part of their official duties; on a worldwide, perpetual, irrevocable, royalty-free basis to BMJ Publishing Group Ltd ("BMJ") its licensees and where the relevant Journal is co-owned by BMJ to the co-owners of the Journal, to publish the Work in this journal and any other BMJ products and to exploit all rights, as set out in our licence.

The Submitting Author accepts and understands that any supply made under these terms is made by BMJ to the Submitting Author unless you are acting as an employee on behalf of your employer or a postgraduate student of an affiliated institution which is paying any applicable article publishing charge ("APC") for Open Access articles. Where the Submitting Author wishes to make the Work available on an Open Access basis (and intends to pay the relevant APC), the terms of reuse of such Open Access shall be governed by a Creative Commons licence – details of these licences and which Creative Commons licence will apply to this Work are set out in our licence referred to above.

Other than as permitted in any relevant BMJ Author's Self Archiving Policies, I confirm this Work has not been accepted for publication elsewhere, is not being considered for publication elsewhere and does not duplicate material already published. I confirm all authors consent to publication of this Work and authorise the granting of this licence.

For peer review only

Does moderate alcohol consumption accelerate the progression of liver disease in NAFLD? A systematic review and narrative synthesis

Helen Jarvis[1] (0000-0001-5039-0228) , Hannah O'Keefe[1] (0000-0002-0107-711X), Dawn Craig[1], Daniel Stow[1], *Barbara Hanratty[1], *Quentin M Anstee[2]

1. Population Health Sciences Institute, Faculty of Medical Sciences, Newcastle University, Newcastle upon Tyne, UK, NE2 4HH

Helen Jarvis helen.jarvis2@ncl.ac.uk
NIHR Doctoral Research Fellow
Hannah O'Keefe hannah.o'keefe@newcastle.ac.uk
Training Fellow in Evidence Synthesis
Dawn Craig (dawn.craig@newcastle.ac.uk)
Professor of Practice in Evidence Synthesis
Daniel Stow (daniel.stow@newcastle.ac.uk)
Research Associate
Barbara Hanratty (barbara.hanratty@newcastle.ac.uk)
Professor of Primary Care and Public Health

2. Translational and Clinical Research institute, Faculty of Medical Sciences, Newcastle University, Newcastle upon Tyne, UK, NE2 4HH

Quentin M Anstee (quentin.anstee@newcastle.ac.uk)
Professor of Experimental Hepatology

* Joint last authorship BH and QMA

1. Correspondence to: Helen Jarvis, GP and NIHR DRF, Population Health Sciences Institute, Faculty of Medical Sciences, Newcastle University, Newcastle upon Tyne, UK, NE2 4HH. Email: helen.jarvis2@ncl.ac.uk

For peer review only

Structured abstract:

Objectives:

Liver disease is a leading cause of premature death, partly driven by the increasing incidence of Non-alcohol related fatty liver disease (NAFLD). Many people with a diagnosis of NAFLD drink moderate amounts of alcohol. There is limited guidance for clinicians looking to advise these patients on the effect this will have on their liver disease progression. This review synthesises the evidence on moderate alcohol consumption and its potential to predict liver disease progression in people with diagnosed NAFLD.

Methods:

A systematic review of longitudinal observational cohort studies was conducted. Databases (MEDLINE, EMBASE, The Cochrane Library and ClinicalTrials.gov) were searched up to September 2020. Studies were included that reported progression of liver disease in adults with NAFLD, looking at moderate levels of alcohol consumption as the exposure of interest. Risk of bias was assessed using the QUIPS tool.

Results:

Of 4241 unique citations, 6 met the inclusion criteria. Pooling of data was not possible due to heterogeneity and studies were analysed using narrative synthesis. Evidence suggested that any level of alcohol consumption is associated with worsening of liver outcomes in NAFLD, even for drinking within recommended limits. Well conducted population based studies estimated up to a doubling of incident liver disease outcomes in NAFLD patients drinking at moderate levels.

Conclusions:

This review found that any level of alcohol intake in NAFLD may be harmful to liver health. Study heterogeneity in definitions of alcohol exposure as well as in outcomes limited quantitative pooling of results. Use of standardised definitions for exposure and outcomes would support future meta-analysis.

Based on this synthesis of the most up to date longitudinal evidence, clinicians seeing patients with NAFLD should currently advise abstinence from alcohol.

Funding and registration:

For peer review only

This study was funded by an NIHR DRF (NIHR300716). The protocol was registered with PROSPERO (#CRD42020168022).

Strengths and limitations of this study:

- This is a timely synthesis of the best available evidence on the role of moderate alcohol consumption in NAFLD
- We used systematic searches to identify literature and prospectively registered our protocol on PROSPERO
- We restricted our inclusion criteria to studies that used longitudinal data to provide evidence of temporal associations
- Due to heterogeneity in definitions of alcohol exposure and outcomes, it was not possible to carry out a meta-analysis
- The existing literature base is limited and only six studies were sufficiently robust to meet our pre-defined inclusion criteria

For peer review only

Main Text:

Introduction:

Liver disease is an increasing health burden across the world, and it is now a major cause of premature (<65 yrs) mortality (1,2). As premature mortality rates from many non-communicable diseases have fallen over the last 30 years, the burden of liver disease is increasing (2,3). The commonest causes of chronic liver disease in high income countries are alcohol related liver disease (ARLD) and metabolic-syndrome related liver disease (or non-alcohol related fatty liver disease - NAFLD). Chronic liver disease is often diagnosed as a result of abnormal liver blood tests or liver imaging, with a fatty liver (steatosis) progressing in some through inflammation (steatohepatitis) and stiffening (fibrosis) to scarring (cirrhosis) increasing the risk of decompensated liver disease or liver cancer. This process of progressive damage to the liver is common to both aetiologies.

Whilst the labelling of liver disease suggests a dichotomy, the clinical reality is that there is significant overlap between ARLD and NAFLD (4). The incidence of obesity and diabetes is rising, and a substantial proportion of the population is drinking alcohol at above recommended

limits (5). It is estimated that up to 17% of the adult population may meet criteria for both NAFLD and ARLD (6). Despite this, there is little guidance available for generalist healthcare professionals, on how to advise people with a diagnosis of NAFLD on safer alcohol consumption.

Recommendations on safe alcohol consumption levels vary worldwide. Increasingly, they take into account the effect that alcohol has on the risk of developing many adverse health outcomes, including cancer. International analysis suggests this should be as low as total abstinence to minimize all health risks (7). Recommended limits for safe alcohol consumption in the UK general population are up to 14 units of alcohol per week in both males and females (8), which equates to 16 grams of alcohol per day at 8g/unit. Moderate alcohol consumption is generally defined in the literature as drinking within, or slightly in excess of, these limits versus complete abstinence (4). There is a significant gap between this recommended 'moderate' limit and the levels of alcohol consumption that would prompt an assessment for alcohol-related liver damage. The UK National Institute of Health and Care Excellence (NICE) recommends offering a liver cirrhosis test to men drinking over 50 units and women drinking over 35 units a week on an ongoing basis over several months (9), leaving a significant proportion who are drinking at and above 14 units a week, but below the levels to have liver assessment based on their alcohol consumption alone. The international differences in definition of how many grams of alcohol a 'unit' contains can create confusion and the reader is directed to table 1 to help in interpreting the study results in the context of UK Government and NICE recommended limits.

Table 1: International definitions of moderate alcohol consumption, UK recommended limits and levels that would warrant assessment for ARLD, all expressed in grams of alcohol and UK units.

Definitions:	grams of alcohol		UK Units of alcohol	
	Daily*	Weekly*	Daily*	Weekly*
Accepted International consensus of moderate alcohol consumption	F: <20	F: <140	F: < 2.5	F: <17.5
	M: <30	M: <210	M: < 3.75	M: < 26.25
UK recommended safe weekly limits	</= 16	</= 112	</= 2	</= 14
NICE thresholds for assessing for liver	F: > 40	F :> 280	F: > 5	F: > 35

For peer review only

cirrhosis	M:> 57	M: >400	M: > 7.1	M: > 50

* Daily and weekly figures are given for comparison only. The bold numbering for each definition is the standard format in which this definition is expressed

There is still uncertainty, and an absence of guidance, on safe levels of alcohol consumption for people with established NAFLD. Indeed, it is not clear that any level of alcohol consumption is safe to minimise progression of the liver disease in this population. It is known that people with very high levels of alcohol consumption (who would meet criteria for a diagnosis of ARLD), and who also have metabolic risk factors, are at even greater risk of adverse liver outcomes (10,11). But there is also some evidence that for people with metabolic risk factors (but who do not have a NAFLD diagnosis), drinking alcohol at low levels may protect against cardiovascular disease, prevent fatty liver disease, and lead to better outcomes than with complete abstinence (12,13). Elucidating the role of alcohol in NAFLD progression is a small part of understanding the interplay of genetic and environmental factors and their effects on the liver; an area of ongoing research and debate (14).

The purpose of this systematic review is to synthesise evidence on the role of moderate alcohol consumption on progression to severe liver disease in people with diagnosed NAFLD. This will help guide the advice given to NAFLD populations around safe alcohol consumption in primary care and specialist settings.

Methods:

The protocol for this review was registered in advance with PROSPERO (International Prospective Register of Systematic Reviews, #CRD42020168022).

Types of studies, inclusion and exclusion criteria

Primary studies were included if they were prospective or retrospective cohort studies. The population of interest was adult patients (>18 years old) with diagnosed NAFLD. The outcome of interest was progression of liver disease in this population. The exposure of interest was no

For peer review only

vs moderate alcohol consumption. For our inclusion criteria we defined ‘moderate consumption’ as up to 35 units per week in females, and 50 units per week in males (levels that would be considered the threshold for definite risk of ARLD according to NICE guidelines (9)). This definition included studies that focused on the effects of alcohol within or just above current weekly recommended limits (the usual definition of ‘moderate’ alcohol consumption), as well as those who looked beyond these levels of consumption, up to the NICE ARLD levels.

Exclusion criteria were as follows: 1) studies where the population had diagnosed ARLD; 2) studies where the population was defined according to their alcohol consumption levels rather than their NAFLD status at baseline; 3) studies where patients already had severe liver disease at the time of cohort entry; 4) cross sectional studies or studies where exposure was only measured at the same time as outcome.

We performed a systematic review following the Preferred Reporting Items for Systematic Reviews and Meta-Analyses (PRISMA) guidelines (15).

Search strategy and data extraction

Potentially relevant studies were identified through systematic literature searches of relevant databases (MEDLINE, EMBASE, The Cochrane Library and ClinicalTrials.gov, CPCI-S (Conference Proceedings Citation Index – Science, Web of Knowledge), OpenGrey (http://www.opengrey.eu/)) in January 2020 and updated in September 2020. No language restrictions were applied, and databases searched documents published from 1990 onwards. Reference lists from potentially relevant papers and previous review articles were hand searched. MeSH (Medical Subject Headings) and free text terms for the NAFLD population, alcohol exposures and liver outcomes of interest were used. Two researchers (HJ and either HO or DS) independently screened titles and abstracts. Any disagreement in full text selection was resolved by consensus. Record screening was also assisted by Rayyan, an online software tool that assesses similarities between selected records and highlights other potentially relevant studies based on the screener’s previous selection (16). Full texts of potentially relevant papers were obtained and read by two independent researchers with reference to the predefined set of criteria to identify final study inclusion. Data were extracted into a standardised form, piloted on 3 studies before full extraction. Data extraction was based on the updated checklist for critical appraisal and data extraction for systematic reviews of prediction studies (CHARMS-PF)

For peer review only

checklist for prognostic studies (17), undertaken by one researcher and checked by a second. Two authors (HJ, HO) assessed the risk of bias independently. Since the included studies were observational cohort studies of prognostic factors, the QUIPS (Quality in Prognostic factor Studies) tool was used (18).

Data synthesis

Pooling of data was not possible due to exposure and outcome heterogeneity across studies. A narrative synthesis (19) was undertaken, with data synthesised by alcohol exposure level. Due to the small number of studies, even those with high risk of bias are included in the synthesis, although this bias assessment is made clear throughout the narrative.

Patient and Public Involvement

Patients and the Public were not involved in the design or conduct of this review but will be involved in the dissemination of findings through a funded PPI steering group and close collaboration with the British Liver Trust.

Results:

The searches identified 4241 unique citations. Of the titles and abstracts screened, 34 articles were selected for full-text screening. Twenty-eight were excluded for reasons reported in the PRISMA diagram (Figure 1). A total of six unique studies representing data from five cohorts were eligible for inclusion in the systematic review, and were assessed for quality (Figure 1), (20-25).

Characteristics of included studies

Further details of included studies are shown in Table 2.

For peer review only

Table 2: Characteristics of included studies

Author /year	Country	Study design and population	Yrs f/u	Method of NAFLD diag*	Method of measuring alcohol consumption	Definition of moderate consumption studied as RF	Study outcomes of interest and event no	Adjustments of interest considered	Adjusted HRs/OR for liver events with 95% CI and *p*-values	Risk of bias
Aberg 2019 (20)	Finland	retrospective data linkage cohort analysis NAFLD population 6462, mean age 53 yrs, 60% M	10.9	FLI >30	Questionnaire at cohort entry	< 50g/day in 10 g categories with abstinence as reference	composite non-fatal and fatal liver disease 58 events	? unclear other than age, sex	per increase in 10g of alcohol per day from abstinence HR 1.43 (1.12 - 1.82) p0.004	high
Aberg 2020 (21)	Finland (FINRISK Health survey)	retrospective data linkage cohort analysis NAFLD population 8345, mean age 53.7 yrs, 60% M	11.1	FLI >60	Questionnaire at cohort entry (recall for past month)	< 50g/day in 10 g categories with abstinence as reference	composite non-fatal and fatal liver disease 152 events	age, sex, smoking, T2DM	g alcohol/day v abstinence<br>0-9 HR 1.38 (0.74-2.58)<br>10-19 HR 2.18(1.04-4.53)<br>20-29 HR 3.62(1.67-7.76)<br>30-39 HR 3.53(1.53-8.14)<br>40-49 HR8.79(3.95-19.56)	low
Ajmera 2018 (22)	USA	retrospective analysis of longitudinal cohorts within NASH CRN NAFLD population 285, mean age 47 yrs, 30% M	3.9	liver biopsy	Questionnaire at cohort entry (Skinner lifetime drinking history)	<2 drinks per day and excluded if >6 drinks on 1 occasion >/= monthly	histological progress on follow up biopsy	age, sex, race, smoking	OR of outcome persistent moderate drinkers v abstinence**<br>resolution of NASH OR 0.32 (0.11-0.92) p0.04<br>fibrosis progression OR 0.00 (-0.29-0.29) p0.99	mod
Chang 2019 (23)	South Korea (Kangbuk Samsung Health Study)	prospective population cohort NAFLD population 58927, mean age 37.7, 82% M	4.9	US	Questionnaire at each study visit (annual or biennial)	10 - 19.9 g/day (F)<br>10 - 29.9 g/day (M)<br>(low 1-9.9g/day)	fibrosis progress as estimated by high indirect serum scores***	age, sex, BMI, smoking, exercise level, education, T2DM, BP	mod v abstinence$ (repeat observations)<br>Fib4: HR 1.33(1.13-1.57)<br>NFS: HR 1.37(1.23-1.52)<br>low v abstinence (repeat observations)<br>Fib 4: HR 1.08(0.91-1.27)	low

For peer review only

									NFS: HR 1.14(1.02-1.27)	
Ekstedt 2009 (24)	Sweden	retrospective cohort NAFLD population 71, mean age 47.3, 72% M	13.8	US and liver biopsy	Questionnaire AUDIT-C and interview at follow up	g/day - no upper limit defined as 'moderate'	fibrosis progress on follow up biopsy	age, sex, BMI, T2DM, fibrosis at baseline	increasing alcohol g/week OR 1.012 (1.000-1.025) p0.055	low
Kawamura 2016 (25)	Japan	prospective cohort NAFLD population 9959, mean age 49, 87% M (included 18 patients >70g alcohol/day defined as ARLD)	5.4	US	Questionnaire at baseline and every 6 months	g/day in categories with <20g/day as reference	HCC on imaging	age, sex, BMI, T2DM, serum markers	g/day alcohol v <20g/day 20-39 HR 0.90(0.11-7.90) p0.919 ≥40-69 HR 2.48(1.01-6.05) p0.047 >70 HR 12.61(5.68-28.00) p0.001	low

Abbreviations: NAFLD = non-alcohol related fatty liver disease; NASH = non-alcohol related steatohepatitis; ARLD = alcohol related liver disease; RF= risk factor; CRN= clinical research network; Yrs = years; M = Male; FLI = Fatty Liver Index; US = hepatic ultrasound; g = grams; HCC = hepatocellular carcinoma; BMI = body mass index; T2DM = type 2 diabetes mellitus; BP = blood pressure; HR = Hazard ratio; OR = Odds ratio; CI = confidence interval; NFS = NAFLD fibrosis score.

**Note multiple differences in means and OR presented for different histological and biochemical outcomes between abstainers, persistent moderate drinkers, and changes in alcohol consumption between biopsies. Presented data represent histological outcomes of potential clinical prognostic significance within the remit of this review.

*** Scores used to estimate fibrosis progression were the Fib4 score, NAFLD fibrosis score (NFS) and APRI score
$ multiple HR presented in paper for different score outcomes for single and repeated outcome measures looking at intermediate/high- or high-risk scores in low and moderate drinkers and different subgroups. Presented data represent outcomes best in keeping with remit of this review using widely used indirect serum markers of liver fibrosis.

For peer review only

For peer review only - http://bmjopen.bmj.com/site/about/guidelines.xhtml

Within the studies meeting inclusion criteria, three (22,23,24) looked at the exposure of alcohol consumption up to, or similar to, the accepted international definition of ‘moderate consumption’. This is <20g/day in women and <30g/day in men (26). Three of the studies (20,21,25) looked at low alcohol consumption but also extended ‘moderate consumption’ up to levels of alcohol consumption which would be considered more consistent with ARLD.

Moderate alcohol consumption (accepted international definitions) and risk of liver disease progression in NAFLD

Three studies examined the effects of alcohol in NAFLD using definitions in keeping with the accepted international definition of ‘moderate consumption’ (22,23,24). Although these studies shared a similar aim, they varied in NAFLD population definition, measurement of alcohol consumption and choice of liver outcomes. Two looked at histological progression outcomes and one used non-invasive indirect blood-based markers of liver fibrosis. Two of the studies were rated as having a low risk of bias (23,24) and one was rated as having a moderate risk (22).

Ajmera et al (22) studied a NAFLD population taken retrospectively from the NASH clinical research network, including populations from an observational study and the placebo arm of two NASH drug trials, all of whom had biopsy proven NAFLD (285 participants). Alcohol consumption was measured at cohort entry and at varying time points up to, and including, follow-up liver biopsy, which occurred, on average, four years later. Multiple histological markers of disease progression were studied, and the authors looked at the association between baseline drinking status and disease progression as well as change in drinking status over time and disease progression. For most of the histological end points studied, there was no significant difference between moderate drinkers and abstainers in outcomes, with the only significant results suggesting that abstainers had less progressive or a higher likelihood of regression of their disease between biopsies, particularly the persistent abstainers. Results should be interpreted in the knowledge that a large number of related histological outcomes were reported, increasing the likelihood of a statistically significant result by chance. The

For peer review only - http://bmjopen.bmj.com/site/about/guidelines.xhtml

absence of detailed information on which other prognostic factors were taken into account, led to a rating of moderate on risk of bias assessment.

A similar study by Ekstedt et al (24) looked at a smaller group (71 participants) of biopsy proven NAFLD, with follow up histology an average of 13.8 years after initial biopsy. Alcohol consumption was assessed at baseline and follow up, with heavy episodic drinking assessed in addition to weekly consumption. Primary outcome was significant fibrosis progression, defined as progression by one or more fibrosis stage or the development of end stage liver disease during follow up. Although higher weekly alcohol consumption showed some tendency to predict fibrosis progression (OR for increase in grams of alcohol per week 1.012 (1.000 - 1.025)) only the presence of heavy episodic drinking (defined as >60g/day in men and >48g/day in women more than once a month) reached statistical significance in predicting fibrosis progression.

Of note in both the Ajmera and Ekstedt studies were the very low levels of alcohol consumption in the ‘moderate drinkers’, with the majority (78%) of the moderate drinkers drinking less than monthly in the Ajmera study and the average weekly alcohol consumption in the Ekstedt study being only 39 g/week. Both studies also included a significant number of patients who already had liver inflammation (NASH) at baseline (over 50% in both studies), indicating a higher proportion of patients with a tendency to progressive disease as compared to a general NAFLD population, as would be expected with biopsy-based studies.

In contrast to the relatively selective biopsy studies, Chang et al (23) studied a large prospective population cohort (Kangbuk Samsung Health Study) of whom 58,927 had ultrasound evidence of fatty liver but without evidence of other liver diagnoses or advanced disease. Alcohol exposure was weekly units at baseline and follow up was for a median of 8.3 years with outcome of interest being progression to advanced liver fibrosis using non-invasive blood-based markers of disease. For moderate drinkers (10 - 30 g/day), the risk of progressing to advanced fibrosis (using intermediate/high Fib4 score as the outcome) was HR 1.33 (1.13 - 1.57), when compared to abstainers. Light drinkers (1-10 g/day) showed a tendency towards more advanced disease when compared to abstainers, but this did not reach statistical significance (HR 1.08 CI 0.91-1.27).

Moderate alcohol consumption (below the threshold that would be consistent with ARLD) and risk of liver disease progression in NAFLD

For peer review only

Three studies extended the definition of moderate alcohol consumption beyond the international consensus definition of ‘moderate consumption’. Two of the studies were rated as having a low risk of bias (21,25), with one rated as high risk of bias (20).

The general population longitudinal data presented by Chang et al (23) is supplemented by two recent related studies by Aberg et al (20,21), using data from the same Finnish National Health Surveys (FINRISK, Health 2000) cohort. The definition of moderate alcohol consumption was increased to include anything up to 50 grams per day in these studies. Although the exposures and outcome measures were the same in the two related studies, the NAFLD population was defined using different fatty liver index (FLI) cut offs values, generating overlapping but distinct study populations. For this reason, data are presented from both studies.
The first study, only available as a conference abstract, (20) used a FLI>30 to retrospectively define their NAFLD population. This low FLI would generally be used as a ‘rule out’ rather than ‘rule in’ cutoff for NAFLD diagnosis (27) and the limited data presented suggests that using abstinence as a reference, any increase in alcohol consumption by 10 g/day, increased incident liver events (combined fatal and non-fatal outcomes) by 43% with a presented HR of 1.43 (1.12 - 1.82) for each 10 g rise in daily alcohol consumption. The data presented contained few details of adjustment factors or analysis plan. This study was graded as having a high risk of bias, and these results should be interpreted with caution.

A larger study (21), based on the same cohort, retrospectively identified a NAFLD population based on a FLI of >60 (the accepted and validated cut off for making a positive diagnosis of NAFLD in the literature (27)). Alcohol intake at cohort entry was based on estimated consumption over the previous year. Lifetime abstainers were used as the reference group. Fatal and non-fatal liver outcomes were studied in 8345 participants over 92 350 person years of follow up. The study concluded that incident liver disease is higher at all levels of alcohol consumption, compared to lifetime abstainers with steadily rising hazard ratios as the level of alcohol consumption increases. Although drinking up to 10g per day was not statistically significantly different to abstaining (HR 1.38 CI 0.74 -2.58 in the final model), levels of alcohol consumption between 10 g and 19 g, which are roughly equivalent to the 14 units per week recommended limits, prognosticated for over double the number of incident liver events in NAFLD patients (HR 2.18 CI 1.05-4.53). At higher levels, which would not necessarily trigger a liver assessment for alcohol related harm in current guidelines, risk of significant liver disease

For peer review only

was nearly nine times higher (for consumption of 40 - 49 g of alcohol a day, HR 8.79 CI 3.95-19.56).

A retrospective Japanese cohort study (25) also looked at stepwise rises in daily alcohol consumption as a prognostic factor for the more specific outcome of hepatocellular carcinoma (HCC) in people with fatty liver (identified on ultrasound). The Kawamura study with 9959 participants followed for a median of nearly 2000 days, had a reference group of people drinking < 20g of alcohol per day, rather than abstainers. This differed from all the other studies reviewed. Only those drinking at between 40-69 g of alcohol a day had a statistically significant increase in rates of HCC (HR 2.48 CI 1.01 - 6.05 p 0.047), with no effect in those drinking at more moderate levels. The population in this retrospective cohort were patients undergoing ultrasound at two tertiary hepatology centres in Japan rather than a general population cohort, and as HCC is known to occur in non-cirrhotic NAFLD (28) comparison with outcomes from other studies should be interpreted with caution.

Excluding the only study rated as having a high risk of bias (20), the other good quality longitudinal studies of varying design, all reported either no association or a negative impact of moderate amounts of alcohol on future liver disease outcomes. This was seen across the studies looking at levels of alcohol consumption within the international definition of moderate consumption, and those that extended this definition of moderate consumption.

Discussion:

Summary of results

In this systematic review of the latest available longitudinal data, we found evidence to suggest that any amount of alcohol, even at low levels, may be harmful for liver health in people with diagnosed NAFLD. This evidence comes from both general population-based cohorts using coded liver outcomes, as well as tertiary centre NAFLD populations defined using histological end points.

Comparison with existing literature

For peer review only

Until recently the majority of evidence in this area has come from cross-sectional studies where alcohol exposure was assessed at the same time as liver outcomes. These data provide somewhat contradictory results, with several studies indicating that moderate alcohol consumption is associated with lower levels of liver disease progression (29,30,31,32) although more recent studies support of our findings, and suggest the opposite (33,34). The design employed in these studies does not allow the assessment of temporal relationships and is open to reverse causality (those with liver damage may be newly abstaining from alcohol for example) in addition to recall and other biases. On the basis of these limitations, cross-sectional studies were excluded from this current review, although they have been widely cited in previous critical reviews in this area, before more recent longitudinal data were available.

In the historical absence of large prospective cohort studies and the impossibility of conducting a controlled trial in the area, comparative work has been undertaken using Mendelian randomisation. This utilises random genetic variations which affects the rate of alcohol metabolism as a proxy measure for alcohol exposure, with randomisation of NAFLD patients based on an allele known to confer lower lifetime alcohol consumption by necessity due to the unpleasant effects of drinking even low levels of alcohol. Findings from this study were supportive of our review, with the group with higher lifetime alcohol consumption showing markers of more severe disease on biopsy, even though alcohol consumption was at very modest levels (35).

In addition to the evidence on the relationship between modest alcohol consumption in NAFLD and liver outcomes, other published studies have focused on overall mortality and cardiovascular outcomes. A study of 4264 participants in an ultrasound diagnosed NAFLD cohort study showed no significant difference in overall mortality in those with alcohol consumption in the low/moderate range versus abstinence after 20 years of follow up (36). A subsequent study with the same US cohort reported a protective effect of low alcohol consumption on overall survival in NAFLD (37). The evidence for a protective effect of low alcohol consumption on cardiovascular outcomes in the general population is generally accepted (38). The evidence for cardiovascular protection in those with NAFLD is more limited, with some evidence that moderate alcohol may provide some benefit (39) but more recent studies finding no protective effects (33,40). The comparative evidence on overall mortality and cardiovascular outcomes highlights the need to assess liver disease risks within these competing contexts.

For peer review only

Strengths and limitations

Although there have been several recent critical reviews of the role of moderate alcohol consumption in NAFLD, the most recent of which reach similar conclusions (4,41,42), these have been wider in their remit with less well-defined inclusion criteria and less systematic methodology. The predetermined inclusion criteria, robust systematic data collection and reporting techniques (in line with PRISMA guidelines) and decision to avoid cross-sectional data are all important in providing the best available evidence to answer the review question of the temporal relationship between moderate alcohol consumption and liver outcomes in NAFLD. The challenges of synthesising observational data, including unmeasured confounding and heterogeneity, were anticipated, but meant that data pooling was not possible.

A particular limitation hindering comparison between studies were the methods of defining moderate alcohol consumption. The consensus for defining a level of alcohol consumption above which a diagnosis of pure NAFLD cannot be made have been supported by the European Association for the Study of the Liver (EASL) and the American Association for the Study of the Liver Diseases (AASLD) and set at 20g/day in women and 30g/day in men (26,43), yet most of the published studies do not use these cut-offs in their data. Until this is standardised across studies, with an additional consensus defining levels above this 'moderate' but not high enough to reach levels associated with a definite diagnosis of ARLD, synthesising the evidence in this area will remain challenging.

Implications for research/practice

This review adds weight to individual studies showing that any level of alcohol intake in NAFLD may be harmful to liver health. Further prospective cohort studies are needed, with detailed definitions/measures of alcohol exposure, and validated clinical liver outcomes, measured at appropriate times. Future research should focus on looking at outcomes in relation to accepted alcohol intake levels used in definitions of NAFLD. It should also take into account that the clinical reality is a dual-aetiology patient who may currently be excluded from both diagnostic categories based on their alcohol intake being too high for NAFLD, and too low for ARLD definitions. This is an ever-expanding patient group seen in many clinical settings.

For peer review only

Based on the evidence synthesised in this review, clinicians seeing patients with NAFLD in primary or secondary care should currently advise abstinence from alcohol to avoid accelerating liver harm. This is likely to be difficult for patients to accept, and public health messaging will need careful thought if it is to have any impact on liver health.

Author contribution statement:

HJ: conceptualization, methodology, conduct, analysis, writing of initial draft
HO: methodology, conduct, figures/infographic, critical review and comments on drafts
DC: supervision of methodology, conduct, critical review and comments on drafts
DS: methodology, conduct, critical review and comments on drafts
BH: conceptualization, supervision of methodology, conduct, analysis, critical review and comments on drafts
QA: conceptualization, supervision of conduct and analysis, critical review and comments on drafts

Competing interest statement:

All authors have completed the Unified Competing Interest form and declare that the study was funded by an NIHR Doctoral Research Fellowship (HJ personal award). There were no financial relationships with any organisations that might have an interest in the submitted work in the previous three years. All authors report no other relationships or activities that could appear to have influenced the submitted work.

HJ reports grants from National Institute for Health research (NIHR), during the conduct of the study; personal fees from Intercept Pharma, personal fees from Norgine , outside the submitted work;
QMA reports grants from European Commission, during the conduct of the study; other from Acuitas Medical, grants, personal fees and other from Allergan/Tobira, other from E3Bio, other from Eli Lilly & Company Ltd, other from Galmed, grants, personal fees and other from Genfit SA, personal fees and other from Gilead, other from Grunthal, other from Imperial Innovations, grants and other from Intercept Pharma Europe Ltd, other from Inventiva, other from Janssen, personal fees from Kenes, other from MedImmune, other from NewGene, grants and other from Pfizer Ltd, other from Raptor Pharma, grants from GlaxoSmithKline, grants and other from Novartis Pharma AG, grants from Abbvie, personal fees from BMS, grants from GSK, other from NGMBio, other from Madrigal, other from Servier, outside the submitted work;

Funding statement:

This work was supported by and NIHR Doctoral Research Fellowship – HJ personal award. Award no: NIHR300716.

For peer review only

Data Sharing statement:

All data relevant to the study are included in the article or uploaded as supplementary information

Ethics approval statement:

Ethical approval was not required for this study as it involved synthesising existing data and did not involve any human or animal participants.

For peer review only

References:

1. Williams R, Aspinall R, Bellis M, et al. Addressing liver disease in the UK: a blueprint for attaining excellence in health care and reducing premature mortality from lifestyle issues of excess consumption of alcohol, obesity, and viral hepatitis. Lancet. 2014;384(9958):1953–97.
2. GBD 2017 Cirrhosis Collaborators. The global, regional, and national burden of cirrhosis by cause in 195 countries and territories, 1990-2017: a systematic analysis for the Global Burden of Disease Study 2017. Lancet Gastroenterol Hepatol. 2020 Mar;5(3):245-266.
3. Martinez R, Lloyd-Sherlock P, Soliz P, et al. Trends in premature avertable mortality from non-communicable diseases for 195 countries and territories, 1990-2017: a population-based study. Lancet Glob Health 2020;8:e511-e523.
4. Boyle M, Masson S, Anstee QM. The bidirectional impacts of alcohol consumption and the metabolic syndrome: Cofactors for progressive fatty liver disease. J Hepatol. 2018;68(2):251–67.
5. Williams R, Alexander G, Armstrong I, et al. Disease burden and costs from excess alcohol consumption, obesity, and viral hepatitis: fourth report of the Lancet Standing Commission on Liver Disease in the UK. Lancet. 2017 Nov 28;
6. Baumeister SE, Alte D, Meyer C, John U. Health Risk drinking and problematic consumption of alcohol in Pomerania: comparative analysis of the Study of Health in Pomerania (SHIP) compared with the Federal German Health and Examination Survey in 1998. Gesundheitswesen. 2005 Jan;67(1):39–47.

7. Collaborators GBDA. Alcohol use and burden for 195 countries and territories, 1990-2016: a systematic analysis for the Global Burden of Disease Study 2016. Lancet 2018;392:1015-1035.
8. UK Chief Medical Officers' Low Risk Drinking Guidelines. Available at: https://assets.publishing.service.gov.uk/government/uploads/system/uploads/attachment_data/file/545937/UK_CMOs__report.pdf
9. Cirrhosis in over 16s: assessment and management | Guidance and guidelines | NICE. Available from: https://www.nice.org.uk/guidance/ng50
10. Hart CL, Morrison DS, Batty GD, Mitchell RJ, Davey Smith G. Effect of body mass index and alcohol consumption on liver disease: analysis of data from two prospective cohort studies. BMJ. 2010 Mar 11;340:c1240.
11. Liu B, Balkwill A, Reeves G, Beral V, Million Women Study Collaborators. Body mass index and risk of liver cirrhosis in middle aged UK women: prospective study. BMJ. 2010 Mar 11;340:c912.
12. Thun MJ, Peto R, Lopez AD, et al. Alcohol consumption and mortality among middle-aged and elderly U.S. adults. N Engl J Med. 1997 Dec 11;337(24):1705–14.
13. Sookoian S, Castaño GO, Pirola CJ. Modest alcohol consumption decreases the risk of non-alcoholic fatty liver disease: a meta-analysis of 43 175 individuals. Gut. 2014 Mar;63(3):530–2.
14. Tarantino G, Citro V, Capone D. Nonalcoholic Fatty Liver Disease: A Challenge from Mechanisms to Therapy. Journal of Clinical Medicine. 2020 Jan;9(1):15. Available from: https://www.mdpi.com/2077-0383/9/1/15
15. Moher D, Liberati A, Tetzlaff J, Altman DG, The PRISMA Group (2009). *Preferred Reporting Items for Systematic Reviews and Meta-Analyses:* The PRISMA Statement. PLoS Med 6(7): e1000097. doi:10.1371/journal.pmed1000097
16. Ouzzani M, Hammady H, Fedorowicz Z, Elmagarmid A. Rayyan-a web and mobile app for systematic reviews. Syst Rev. 2016 05;5(1):210.
17. Moons KGM, Groot JAH de, Bouwmeester W, et al. Critical Appraisal and Data Extraction for Systematic Reviews of Prediction Modelling Studies: The CHARMS Checklist. PLOS Medicine. 2014 Oct 14;11(10):e1001744.
18. Hayden JA, van der Windt DA, Cartwright JL, Côté P, Bombardier C. Assessing bias in studies of prognostic factors. Ann Intern Med. 2013 Feb 19;158(4):280–6.

For peer review only - http://bmjopen.bmj.com/site/about/guidelines.xhtml

19. Popay J, Roberts H, Sowden A, et al. Guidance on the conduct of narrative synthesis in systematic reviews: a product from the ESRC Methods Programme. Lancaster: Lancaster University. 2006;10(2.1):1018–4643
20. Åberg F, Puukka P, Sahlman P et al. In NAFLD, alcohol drinking habits and genetics predict progression to advanced liver disease: follow-up of population surveys. Journal of Hepatology. 2019; 70: e141. (poster abstract)
21. Åberg F, Puukka P, Salomaa V, et al. Risks of Light and Moderate Alcohol Use in Fatty Liver Disease: Follow-Up of Population Cohorts. Hepatology. 2020 Mar;71(3):835–48.
22. Ajmera V, Belt P, Wilson LA, et al. Among Patients With Nonalcoholic Fatty Liver Disease, Modest Alcohol Use Is Associated With Less Improvement in Histologic Steatosis and Steatohepatitis. Clin Gastroenterol Hepatol. 2018;16(9):1511-1520.e5.
23. Chang Y, Cho YK, Kim Y, et al. Nonheavy Drinking and Worsening of Noninvasive Fibrosis Markers in Nonalcoholic Fatty Liver Disease: A Cohort Study. Hepatology. 2019;69(1):64–75.
24. Ekstedt M, Franzén LE, Holmqvist M, et al. Alcohol consumption is associated with progression of hepatic fibrosis in non-alcoholic fatty liver disease. Scand J Gastroenterol. 2009;44(3):366–74.
25. Kawamura Y, Arase Y, Ikeda K, et al. Effects of Alcohol Consumption on Hepatocarcinogenesis in Japanese Patients With Fatty Liver Disease. Clin Gastroenterol Hepatol. 2016 Apr;14(4):597–605.
26. European Association for the Study of the Liver (EASL), European Association for the Study of Diabetes (EASD), European Association for the Study of Obesity (EASO). EASL-EASD-EASO Clinical Practice Guidelines for the Management of Non-Alcoholic Fatty Liver Disease. Obes Facts. 2016;9(2):65–90.
27. Bedogni G, Bellentani S, Miglioli L, et al. The Fatty Liver Index: a simple and accurate predictor of hepatic steatosis in the general population. BMC Gastroenterol. 2006 Nov 2;6:33.
28. Negro F. Natural history of NASH and HCC. Liver Int. 2020;40 Suppl 1:72–6.
29. Dunn W, Sanyal AJ, Brunt EM, et al. Modest alcohol consumption is associated with decreased prevalence of steatohepatitis in patients with non-alcoholic fatty liver disease (NAFLD). J Hepatol. 2012 Aug;57(2):384–91.
30. Hagström H, Nasr P, Ekstedt M, et al. Low to moderate lifetime alcohol consumption is associated with less advanced stages of fibrosis in non-alcoholic fatty liver disease. Scand J Gastroenterol. 2017 Feb;52(2):159–65.

For peer review only

31. Kwon HK, Greenson JK, Conjeevaram HS. Effect of lifetime alcohol consumption on the histological severity of non-alcoholic fatty liver disease. Liver Int. 2014 Jan;34(1):129–35.
32. Mitchell T, Jeffrey GP, MacQuillan GC, et al. The impact of alcohol consumption on hepatic fibrosis in non-alcoholic fatty liver disease. Hepatology. 2016. Vol 64 (supp) 548A (poster abstract)
33. Kashiwagi K, Yamaguchi A, Shiba S, et al. Moderate alcohol consumption is not associated with subclinical cardiovascular damage but with hepatic fibrosis in non-alcoholic fatty liver disease. Alcohol. 2020 Jul 30;89:1–7.
34. Mulazzani L, Alvisi M, Tovoli F, et al. Moderate alcohol consumption is associated with risk of fibrosis in patients with non-alcoholic fatty liver disease.Digestive and Liver Disease. 2020; 52 (0): pp e43-e44.
35. Sookoian S, Flichman D, Castaño GO, Pirola CJ. Mendelian randomisation suggests no beneficial effect of moderate alcohol consumption on the severity of nonalcoholic fatty liver disease. Aliment Pharmacol Ther. 2016;44(11–12):1224–34.
36. Younossi ZM, Stepanova M, Ong J, et al. Effects of Alcohol Consumption and Metabolic Syndrome on Mortality in Patients With Nonalcoholic and Alcohol-Related Fatty Liver Disease. Clin Gastroenterol Hepatol. 2018 Nov 23;
37. Hajifathalian K, Torabi Sagvand B, McCullough AJ. Effect of Alcohol Consumption on Survival in Nonalcoholic Fatty Liver Disease: A National Prospective Cohort Study. Hepatology. 2019;70(2):511–21.
38. Klatsky AL. Alcohol and cardiovascular diseases. Expert Review of Cardiovascular Therapy [Internet]. 2009 May 1 [cited 2020 Sep 14];7(5):499–506.
39. Sinn DH, Gwak G-Y, Cho J, et al. Modest alcohol consumption and carotid plaques or carotid artery stenosis in men with non-alcoholic fatty liver disease. Atherosclerosis. 2014 Jun 1;234(2):270–5.
40. VanWagner LB, Ning H, Allen NB, et al. Alcohol Use and Cardiovascular Disease Risk in Patients With Nonalcoholic Fatty Liver Disease. Gastroenterology. 2017;153(5):1260-1272.e3.
41. Ajmera VH, Terrault NA, Harrison SA. Is moderate alcohol use in nonalcoholic fatty liver disease good or bad? A critical review. Hepatology. 2017 Jun;65(6):2090–9.
42. Åberg F, Färkkilä M, Männistö V. Interaction Between Alcohol Use and Metabolic Risk Factors for Liver Disease: A Critical Review of Epidemiological Studies. Alcohol Clin Exp Res. 2020;44(2):384–403.

For peer review only

43. Chalasani N, Younossi Z, Lavine JE, et al. The diagnosis and management of non-alcoholic fatty liver disease: practice guideline by the American Gastroenterological Association, American Association for the Study of Liver Diseases, and American College of Gastroenterology. Gastroenterology. 2012 Jun;142(7):1592–609.

Figure 1: PRISMA diagram of study selection

For peer review only

Fig 1: PRISMA diagram of study selection

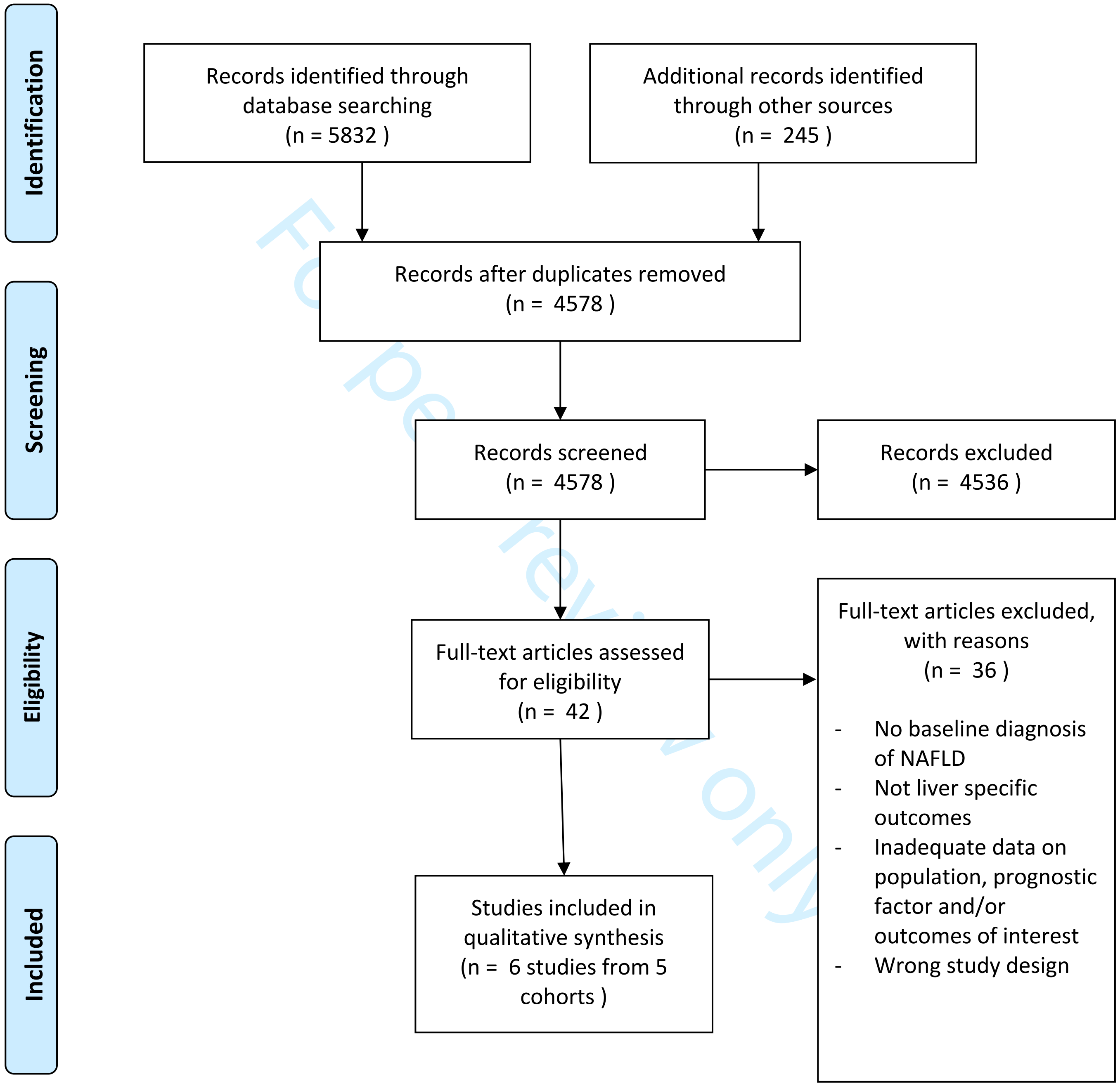

For peer review only

PRISMA 2009 Checklist

Section/topic	#	Checklist item	Reported on page #
**TITLE**			
Title	1	Identify the report as a systematic review, meta-analysis, or both.	Within title
**ABSTRACT**			
Structured summary	2	Provide a structured summary including, as applicable: background; objectives; data sources; study eligibility criteria, participants, and interventions; study appraisal and synthesis methods; results; limitations; conclusions and implications of key findings; systematic review registration number.	Abstract section para 1-4
**INTRODUCTION**			
Rationale	3	Describe the rationale for the review in the context of what is already known.	Introduction Para 1-3
Objectives	4	Provide an explicit statement of questions being addressed with reference to participants, interventions, comparisons, outcomes, and study design (PICOS).	Introduction Para 4
**METHODS**			
Protocol and registration	5	Indicate if a review protocol exists, if and where it can be accessed (e.g., Web address), and, if available, provide registration information including registration number.	Methods Para 1
Eligibility criteria	6	Specify study characteristics (e.g., PICOS, length of follow-up) and report characteristics (e.g., years considered, language, publication status) used as criteria for eligibility, giving rationale.	Methods – para 2-3
Information sources	7	Describe all information sources (e.g., databases with dates of coverage, contact with study authors to identify additional studies) in the search and date last searched.	Methods Para 3-4
Search	8	Present full electronic search strategy for at least one database, including any limits used, such that it could be repeated.	On request
Study selection	9	State the process for selecting studies (i.e., screening, eligibility, included in systematic review, and, if applicable, included in the meta-analysis).	Methods Para 2 Results: Fig 1
Data collection process	10	Describe method of data extraction from reports (e.g., piloted forms, independently, in duplicate) and any processes for obtaining and confirming data from investigators.	Methods Para 3,4
Data items	11	List and define all variables for which data were sought (e.g., PICOS, funding sources) and any assumptions and simplifications made.	Methods Para 2-3

For peer review only

PRISMA 2009 Checklist

Risk of bias in individual studies	12	Describe methods used for assessing risk of bias of individual studies (including specification of whether this was done at the study or outcome level), and how this information is to be used in any data synthesis.	Methods para 4-5
Summary measures	13	State the principal summary measures (e.g., risk ratio, difference in means).	N/A
Synthesis of results	14	Describe the methods of handling data and combining results of studies, if done, including measures of consistency (e.g., I^2) for each meta-analysis.	N/A

Section/topic	#	Checklist item	Reported on page #
Risk of bias across studies	15	Specify any assessment of risk of bias that may affect the cumulative evidence (e.g., publication bias, selective reporting within studies).	N/A
Additional analyses	16	Describe methods of additional analyses (e.g., sensitivity or subgroup analyses, meta-regression), if done, indicating which were pre-specified.	N/A
**RESULTS**			
Study selection	17	Give numbers of studies screened, assessed for eligibility, and included in the review, with reasons for exclusions at each stage, ideally with a flow diagram.	Results Fig 1
Study characteristics	18	For each study, present characteristics for which data were extracted (e.g., study size, PICOS, follow-up period) and provide the citations.	Results Table 1
Risk of bias within studies	19	Present data on risk of bias of each study and, if available, any outcome level assessment (see item 12).	Results Table 1
Results of individual studies	20	For all outcomes considered (benefits or harms), present, for each study: (a) simple summary data for each intervention group (b) effect estimates and confidence intervals, ideally with a forest plot.	a) Table 1 b) N/A
Synthesis of results	21	Present results of each meta-analysis done, including confidence intervals and measures of consistency.	N/A
Risk of bias across studies	22	Present results of any assessment of risk of bias across studies (see Item 15).	N/A
Additional analysis	23	Give results of additional analyses, if done (e.g., sensitivity or subgroup analyses, meta-regression [see Item 16]).	N/A
**DISCUSSION**			
Summary of evidence	24	Summarize the main findings including the strength of evidence for each main outcome; consider their relevance to key groups (e.g., healthcare providers, users, and policy makers).	Discussion Para 1-3
Limitations	25	Discuss limitations at study and outcome level (e.g., risk of bias), and at review-level (e.g., incomplete retrieval of identified research, reporting bias).	Discussion para 5-6
Conclusions	26	Provide a general interpretation of the results in the context of other evidence, and implications for future research.	Discussion para 2,3,4 7,8

For peer review only

For peer review only - http://bmjopen.bmj.com/site/about/guidelines.xhtml

PRISMA 2009 Checklist

**FUNDING**			
Funding	27	Describe sources of funding for the systematic review and other support (e.g., supply of data); role of funders for the systematic review.	Title page

From: Moher D, Liberati A, Tetzlaff J, Altman DG, The PRISMA Group (2009). Preferred Reporting Items for Systematic Reviews and Meta-Analyses: The PRISMA Statement. PLoS Med 6(7): e1000097. doi:10.1371/journal.pmed1000097

For more information, visit: **www.prisma-statement.org**.

Page 2 of 2

For peer review only

BMJ Open

Does moderate alcohol consumption accelerate the progression of liver disease in NAFLD? A systematic review and narrative synthesis

Journal:	*BMJ Open*
Manuscript ID	bmjopen-2021-049767.R2
Article Type:	Original research
Date Submitted by the Author:	18-Nov-2021

Complete List of Authors:	Jarvis, Helen; Newcastle University, Population Health Sciences Institute<br>O'Keefe, Hannah; Newcastle University, Population Health Sciences Institute<br>Craig, Dawn; Newcastle University, Population Health Sciences Institute<br>Stow, Daniel; Newcastle University, Population Health Sciences Institute<br>Hanratty, Barbara; Newcastle University, Population Health Sciences Institute<br>Anstee, Quentin; Newcastle University, Translational and Clinical Research Institute

**Primary Subject Heading**:	Gastroenterology and hepatology
Secondary Subject Heading:	Epidemiology, General practice / Family practice
Keywords:	Hepatology < INTERNAL MEDICINE, GENERAL MEDICINE (see Internal Medicine), PRIMARY CARE

SCHOLARONE™
Manuscripts

I, the Submitting Author has the right to grant and does grant on behalf of all authors of the Work (as defined in the below author licence), an exclusive licence and/or a non-exclusive licence for contributions from authors who are: i) UK Crown employees; ii) where BMJ has agreed a CC-BY licence shall apply, and/or iii) in accordance with the terms applicable for US Federal Government officers or employees acting as part of their official duties; on a worldwide, perpetual, irrevocable, royalty-free basis to BMJ Publishing Group Ltd ("BMJ") its licensees and where the relevant Journal is co-owned by BMJ to the co-owners of the Journal, to publish the Work in this journal and any other BMJ products and to exploit all rights, as set out in our licence.

The Submitting Author accepts and understands that any supply made under these terms is made by BMJ to the Submitting Author unless you are acting as an employee on behalf of your employer or a postgraduate student of an affiliated institution which is paying any applicable article publishing charge ("APC") for Open Access articles. Where the Submitting Author wishes to make the Work available on an Open Access basis (and intends to pay the relevant APC), the terms of reuse of such Open Access shall be governed by a Creative Commons licence – details of these licences and which Creative Commons licence will apply to this Work are set out in our licence referred to above.

Other than as permitted in any relevant BMJ Author's Self Archiving Policies, I confirm this Work has not been accepted for publication elsewhere, is not being considered for publication elsewhere and does not duplicate material already published. I confirm all authors consent to publication of this Work and authorise the granting of this licence.

For peer review only

Does moderate alcohol consumption accelerate the progression of liver disease in NAFLD? A systematic review and narrative synthesis

Helen Jarvis[1] (0000-0001-5039-0228) , Hannah O'Keefe[1] (0000-0002-0107-711X), Dawn Craig[1], Daniel Stow[1], *Barbara Hanratty[1], *Quentin M Anstee[2]

1. Population Health Sciences Institute, Faculty of Medical Sciences, Newcastle University, Newcastle upon Tyne, UK, NE2 4HH

Helen Jarvis helen.jarvis2@ncl.ac.uk
NIHR Doctoral Research Fellow
Hannah O'Keefe hannah.o'keefe@newcastle.ac.uk
Training Fellow in Evidence Synthesis
Dawn Craig (dawn.craig@newcastle.ac.uk)
Professor of Practice in Evidence Synthesis
Daniel Stow (daniel.stow@newcastle.ac.uk)
Research Associate
Barbara Hanratty (barbara.hanratty@newcastle.ac.uk)
Professor of Primary Care and Public Health

2. Translational and Clinical Research institute, Faculty of Medical Sciences, Newcastle University, Newcastle upon Tyne, UK, NE2 4HH

Quentin M Anstee (quentin.anstee@newcastle.ac.uk)
Professor of Experimental Hepatology

* Joint last authorship BH and QMA

1. Correspondence to: Helen Jarvis, GP and NIHR DRF, Population Health Sciences Institute, Faculty of Medical Sciences, Newcastle University, Newcastle upon Tyne, UK, NE2 4HH. Email: helen.jarvis2@ncl.ac.uk

For peer review only

Structured abstract:

Objectives:

Liver disease is a leading cause of premature death, partly driven by the increasing incidence of Non-alcohol related fatty liver disease (NAFLD). Many people with a diagnosis of NAFLD drink moderate amounts of alcohol. There is limited guidance for clinicians looking to advise these patients on the effect this will have on their liver disease progression. This review synthesises the evidence on moderate alcohol consumption and its potential to predict liver disease progression in people with diagnosed NAFLD.

Methods:

A systematic review of longitudinal observational cohort studies was conducted. Databases (MEDLINE, EMBASE, The Cochrane Library and ClinicalTrials.gov) were searched up to September 2020. Studies were included that reported progression of liver disease in adults with NAFLD, looking at moderate levels of alcohol consumption as the exposure of interest. Risk of bias was assessed using the QUIPS tool.

Results:

Of 4578 unique citations, 6 met the inclusion criteria. Pooling of data was not possible due to heterogeneity and studies were analysed using narrative synthesis. Evidence suggested that any level of alcohol consumption is associated with worsening of liver outcomes in NAFLD, even for drinking within recommended limits. Well conducted population based studies estimated up to a doubling of incident liver disease outcomes in NAFLD patients drinking at moderate levels.

Conclusions:

This review found that any level of alcohol intake in NAFLD may be harmful to liver health. Study heterogeneity in definitions of alcohol exposure as well as in outcomes limited quantitative pooling of results. Use of standardised definitions for exposure and outcomes would support future meta-analysis.

Based on this synthesis of the most up to date longitudinal evidence, clinicians seeing patients with NAFLD should currently advise abstinence from alcohol.

Funding and registration:

For peer review only

This study was funded by an NIHR DRF (NIHR300716). The protocol was registered with PROSPERO (#CRD42020168022).

Strengths and limitations of this study:

- This is a timely synthesis of the best available evidence on the role of moderate alcohol consumption in NAFLD
- We used systematic searches to identify literature and prospectively registered our protocol on PROSPERO
- We restricted our inclusion criteria to studies that used longitudinal data to provide evidence of temporal associations
- Due to heterogeneity in definitions of alcohol exposure and outcomes, it was not possible to carry out a meta-analysis
- The existing literature base is limited and only six studies were sufficiently robust to meet our pre-defined inclusion criteria

For peer review only

Main Text:

Introduction:

Liver disease is an increasing health burden across the world, and it is now a major cause of premature (<65 yrs) mortality (1,2). As premature mortality rates from many non-communicable diseases have fallen over the last 30 years, the burden of liver disease is increasing (2,3). The commonest causes of chronic liver disease in high income countries are alcohol related liver disease (ARLD) and metabolic-syndrome related liver disease (or non-alcohol related fatty liver disease - NAFLD). Chronic liver disease is often diagnosed as a result of abnormal liver blood tests or liver imaging, with a fatty liver (steatosis) progressing in some through inflammation (steatohepatitis) and stiffening (fibrosis) to scarring (cirrhosis) increasing the risk of decompensated liver disease or liver cancer. This process of progressive damage to the liver is common to both aetiologies.

Whilst the labelling of liver disease suggests a dichotomy, the clinical reality is that there is significant overlap between ARLD and NAFLD (4). The incidence of obesity and diabetes is rising, and a substantial proportion of the population is drinking alcohol at above recommended

limits (5). It is estimated that up to 17% of the adult population may meet criteria for both NAFLD and ARLD (6). Despite this, there is little guidance available for generalist healthcare professionals, on how to advise people with a diagnosis of NAFLD on safer alcohol consumption.

Recommendations on safe alcohol consumption levels vary worldwide. Increasingly, they take into account the effect that alcohol has on the risk of developing many adverse health outcomes, including cancer. International analysis suggests this should be as low as total abstinence to minimize all health risks (7). Recommended limits for safe alcohol consumption in the UK general population are up to 14 units of alcohol per week in both males and females (8), which equates to 16 grams of alcohol per day at 8g/unit. Moderate alcohol consumption is generally defined in the literature as drinking within, or slightly in excess of, these limits versus complete abstinence (4). There is a significant gap between this recommended ‘moderate’ limit and the levels of alcohol consumption that would prompt an assessment for alcohol-related liver damage. The UK National Institute of Health and Care Excellence (NICE) recommends offering a liver cirrhosis test to men drinking over 50 units and women drinking over 35 units a week on an ongoing basis over several months (9), leaving a significant proportion who are drinking at and above 14 units a week, but below the levels to have liver assessment based on their alcohol consumption alone. The international differences in definition of how many grams of alcohol a ‘unit’ contains can create confusion and the reader is directed to table 1 to help in interpreting the study results in the context of UK Government and NICE recommended limits.

For peer review only

Table 1: International definitions of moderate alcohol consumption, UK recommended limits and levels that would warrant assessment for ARLD, all expressed in grams of alcohol and UK units.

Definitions:	grams of alcohol		UK Units of alcohol	
	Daily*	Weekly*	Daily*	Weekly*
Accepted International consensus of moderate alcohol consumption	F: <20	F: <140	F: < 2.5	F: <17.5
	M: <30	M: <210	M: < 3.75	M: < 26.25
UK recommended safe weekly limits	</= 16	</= 112	</= 2	</= 14
NICE thresholds for assessing for liver	F: > 40	F :> 280	F: > 5	F: > 35

For peer review only - http://bmjopen.bmj.com/site/about/guidelines.xhtml

cirrhosis	M:> 57	M: >400	M: > 7.1	M: > 50

* Daily and weekly figures are given for comparison only. The bold numbering for each definition is the standard format in which this definition is expressed

There is still uncertainty, and an absence of guidance, on safe levels of alcohol consumption for people with established NAFLD. Indeed, it is not clear that any level of alcohol consumption is safe to minimise progression of the liver disease in this population. It is known that people with very high levels of alcohol consumption (who would meet criteria for a diagnosis of ARLD), and who also have metabolic risk factors, are at even greater risk of adverse liver outcomes (10,11). But there is also some evidence that for people with metabolic risk factors (but who do not have a NAFLD diagnosis), drinking alcohol at low levels may protect against cardiovascular disease, prevent fatty liver disease, and lead to better outcomes than with complete abstinence (12,13). Elucidating the role of alcohol in NAFLD progression is a small part of understanding the interplay of genetic and environmental factors and their effects on the liver; an area of ongoing research and debate (14).

The purpose of this systematic review is to synthesise evidence on the role of moderate alcohol consumption on progression to severe liver disease in people with diagnosed NAFLD. This will help guide the advice given to NAFLD populations around safe alcohol consumption in primary care and specialist settings.

Methods:

The protocol for this review was registered in advance with PROSPERO (International Prospective Register of Systematic Reviews, #CRD42020168022).

Types of studies, inclusion and exclusion criteria

Primary studies were included if they were prospective or retrospective cohort studies. The population of interest was adult patients (>18 years old) with diagnosed NAFLD. The outcome of interest was progression of liver disease in this population. The exposure of interest was no

For peer review only

vs moderate alcohol consumption. For our inclusion criteria we defined ‘moderate consumption’ as up to 35 units per week in females, and 50 units per week in males (levels that would be considered the threshold for definite risk of ARLD according to NICE guidelines (9)). This definition included studies that focused on the effects of alcohol within or just above current weekly recommended limits (the usual definition of ‘moderate’ alcohol consumption), as well as those who looked beyond these levels of consumption, up to the NICE ARLD levels.

Exclusion criteria were as follows: 1) studies where the population had diagnosed ARLD; 2) studies where the population was defined according to their alcohol consumption levels rather than their NAFLD status at baseline; 3) studies where patients already had severe liver disease at the time of cohort entry; 4) cross sectional studies or studies where exposure was only measured at the same time as outcome.

We performed a systematic review following the Preferred Reporting Items for Systematic Reviews and Meta-Analyses (PRISMA) guidelines (15).

Search strategy and data extraction

Potentially relevant studies were identified through systematic literature searches of relevant databases (MEDLINE, EMBASE, The Cochrane Library and ClinicalTrials.gov, CPCI-S (Conference Proceedings Citation Index – Science, Web of Knowledge), CINAHL(EBSCO)) in January 2020 and updated in September 2020. No language restrictions were applied, and databases searched documents published from 1990 onwards. Reference lists from potentially relevant papers and previous review articles were hand searched. MeSH (Medical Subject Headings) and free text terms for the NAFLD population, alcohol exposures and liver outcomes of interest were used. Two researchers (HJ and either HO or DS) independently screened titles and abstracts. Any disagreement in full text selection was resolved by consensus. Record screening was also assisted by Rayyan, an online software tool that assesses similarities between selected records and highlights other potentially relevant studies based on the screener’s previous selection (16). Full texts of potentially relevant papers were obtained and read by two independent researchers with reference to the predefined set of criteria to identify final study inclusion. Data were extracted into a standardised form, piloted on 3 studies before full extraction. Data extraction was based on the updated checklist for critical appraisal and data extraction for systematic reviews of prediction studies (CHARMS-PF) checklist for prognostic

For peer review only

studies (17), undertaken by one researcher and checked by a second. Two authors (HJ, HO) assessed the risk of bias independently. Since the included studies were observational cohort studies of prognostic factors, the QUIPS (Quality in Prognostic factor Studies) tool was used (18).

Data synthesis

Pooling of data was not possible due to exposure and outcome heterogeneity across studies. A narrative synthesis (19) was undertaken, with data synthesised by alcohol exposure level. Due to the small number of studies, even those with high risk of bias are included in the synthesis, although this bias assessment is made clear throughout the narrative.

Patient and Public Involvement

Patients and the Public were not involved in the design or conduct of this review but will be involved in the dissemination of findings through a funded PPI steering group and close collaboration with the British Liver Trust.

Results:

The searches identified 4578 unique citations. Of the titles and abstracts screened, 42 articles were selected for full-text screening. Thirty six were excluded at this stage for reasons summarised in the PRISMA diagram (Figure 1). In seven of the excluded studies the population did not have a baseline diagnosis of NAFLD (20,21,22,23,24,25,26) and in five studies the population already had advanced liver disease at baseline (27,28,29,30,31). Five of the excluded studied focused on non liver specific outcomes such as overall mortality (32,33,34,35,36), while eleven were conference abstracts or short papers which held inadequate data on either population , exposure or outcomes (20,21,23,25,29,32,33,37,38,39,40). The commonest reason for exclusion at full text stage was study design, mainly cross sectional studies looking at a single time point to assess exposure and outcome (24,30,31,37,38,39,41,42,43,44,45,46,47). There were also eight studies which on full text reading were review articles or editorials (48,49,50,51,52,53,54,55). A total of six

For peer review only

unique studies representing data from five cohorts were eligible for inclusion in the systematic review, and were assessed for quality (Figure 1), (56-61).

Characteristics of included studies

Further details of included studies are shown in Table 2.

For peer review only

Table 2: Characteristics of included studies

**Author /year**	**Country**	**Study design and population**	**Yrs f/u**	**Method of NAFLD diag***	**Method of measuring alcohol consumption**	**Definition of moderate consumption studied as RF**	**Study outcomes of interest and event no**	**Adjustments of interest considered**	**Adjusted HRs/OR/ mean differences for liver events with 95% CI and *p*-values**	**Risk of bias**
Aberg 2019 (56)	Finland	retrospective data linkage cohort analysis NAFLD population 6462, mean age 53 yrs, 60% M	10.9	FLI >30	Questionnaire at cohort entry	< 50g/day in 10 g categories with abstinence as reference	composite non-fatal and fatal liver disease 58 events	? unclear other than age, sex	per increase in 10g of alcohol per day v abstinence HR 1.43 (1.12 - 1.82) p=0.004	high
Aberg 2020 (57)	Finland (FINRISK Health survey)	retrospective data linkage cohort analysis NAFLD population 8345, mean age 53.7 yrs, 60% M	11.1	FLI >60	Questionnaire at cohort entry (recall for past month)	< 50g/day in 10 g categories with abstinence as reference	composite non-fatal and fatal liver disease 152 events	age, sex, smoking, T2DM	g alcohol/day v abstinence<br>0-9 HR 1.38 (0.74-2.58)<br>10-19 HR 2.18(1.04-4.53)<br>20-29 HR 3.62(1.67-7.76)<br>30-39 HR 3.53(1.53-8.14)<br>40-49 HR8.79(3.95-19.56)	low
Ajmera 2018 (58)	USA	retrospective analysis of longitudinal cohorts within NASH CRN NAFLD population 285, mean age 47 yrs, 30% M	3.9	liver biopsy	Questionnaire at cohort entry (Skinner lifetime drinking history)	<2 drinks per day and excluded if >6 drinks on 1 occasion >/= monthly	histological resolution or progression on follow up biopsy	age, sex, race, smoking	persistent moderate drinkers v abstinence**<br>**resolution** of NASH: OR 0.32 (0.11-0.92) p =0.04<br>fibrosis progression: adj mean diff 0.00 (-0.29-0.29) p =0.99	mod
Chang 2019 (59)	South Korea (Kangbuk Samsung Health Study)	prospective population cohort<br>NAFLD population 58927, mean age 37.7, 82% M	4.9	US	Questionnaire at each study visit (annual or biennial)	10 - 19.9 g/day (F)<br>10 - 29.9 g/day (M)<br>(low 1-9.9g/day)	fibrosis progress as estimated by high indirect serum scores***	age, sex, BMI, smoking, exercise level, education, T2DM, BP	mod v abstinence$<br>(repeat observations)<br>Fib4: HR 1.33(1.13-1.57)<br>NFS: HR 1.37(1.23-1.52)<br>low v abstinence<br>(repeat observations)<br>Fib 4: HR 1.08(0.91-1.27)	low

For peer review only

									NFS: HR 1.14(1.02-1.27)	
Ekstedt 2009 (60)	Sweden	retrospective cohort NAFLD population 71, mean age 47.3, 72% M	13.8	US and liver biopsy	Questionnaire AUDIT-C and interview at follow up	g/day - no upper limit defined as 'moderate'	fibrosis progress on follow up biopsy	age, sex, BMI, T2DM, fibrosis at baseline	increasing alcohol g/week v abstinence OR 1.012 (1.000-1.025) p = 0.055	low
Kawamura 2016 (61)	Japan	prospective cohort NAFLD population 9959, mean age 49, 87% M (included 18 patients >70g alcohol/day defined as ARLD)	5.4	US	Questionnaire at baseline and every 6 months	g/day in categories with <20g/day as reference	HCC on imaging	age, sex, BMI, T2DM, serum markers	g/day alcohol v <20g/day 20-39 HR 0.90(0.11-7.90) p=0.919 ≥40-69 HR 2.48(1.01-6.05) p=0.047 >70 HR 12.61(5.68-28.00) p=0.001	low

Abbreviations: NAFLD = non-alcohol related fatty liver disease; NASH = non-alcohol related steatohepatitis; ARLD = alcohol related liver disease; RF= risk factor; CRN= clinical research network; Yrs = years; M = Male; FLI = Fatty Liver Index; US = hepatic ultrasound; g = grams; HCC = hepatocellular carcinoma; BMI = body mass index; T2DM = type 2 diabetes mellitus; BP = blood pressure; HR = Hazard ratio; OR = Odds ratio; CI = confidence interval; NFS = NAFLD fibrosis score.

**Note multiple differences in means and OR presented for different histological and biochemical outcomes between abstainers, persistent moderate drinkers, and changes in alcohol consumption between biopsies. Presented data represent histological outcomes of potential clinical prognostic significance within the remit of this review comparing persistent moderate drinking to abstinence

*** Scores used to estimate fibrosis progression were the Fib4 score, NAFLD fibrosis score (NFS) and APRI score
$ multiple HR presented in paper for different score outcomes for single and repeated outcome measures looking at intermediate/high- or high-risk scores in low and moderate drinkers and different subgroups. Presented data represent outcomes best in keeping with remit of this review using widely used indirect serum markers of liver fibrosis.

For peer review only

Within the studies meeting inclusion criteria, three (58,59,60) looked at the exposure of alcohol consumption up to, or similar to, the accepted international definition of 'moderate consumption'. This is <20g/day in women and <30g/day in men (26). Three of the studies (56,57,61) looked at low alcohol consumption but also extended 'moderate consumption' up to levels of alcohol consumption which would be considered more consistent with ARLD.

Moderate alcohol consumption (accepted international definitions) and risk of liver disease progression in NAFLD

Three studies examined the effects of alcohol in NAFLD using definitions in keeping with the accepted international definition of 'moderate consumption' (58,59,60). Although these studies shared a similar aim, they varied in NAFLD population definition, measurement of alcohol consumption and choice of liver outcomes. Two looked at histological progression outcomes and one used non-invasive indirect blood-based markers of liver fibrosis. Two of the studies were rated as having a low risk of bias (59,60) and one was rated as having a moderate risk (58).

Ajmera et al (58) studied a NAFLD population taken retrospectively from the NASH clinical research network, including populations from an observational study and the placebo arm of two NASH drug trials, all of whom had biopsy proven NAFLD (285 participants). Alcohol consumption was measured at cohort entry and at varying time points up to, and including, follow-up liver biopsy, which occurred, on average, 3.9 years later. Multiple histological markers of disease progression and resolution were studied, and the authors looked at the association between baseline drinking status and disease, as well as change in drinking status over time and disease progression/resolution. For most of the histological end points studied, there was no significant difference between moderate drinkers and abstainers in outcomes, with the only significant results suggesting that abstainers had less progressive or a higher likelihood of resolution of their disease between biopsies, particularly the persistent abstainers when compared to the persistent moderate drinkers. Results should be interpreted in the knowledge that a large number of related histological outcomes were reported, increasing the likelihood of a statistically significant result by chance. The study also had a relatively short follow up period

For peer review only

between biopsies. The absence of detailed information on which other prognostic factors were taken into account, led to a rating of moderate on risk of bias assessment.

A similar study by Ekstedt et al (60) looked at a smaller group (71 participants) of biopsy proven NAFLD, with follow up histology an average of 13.8 years after initial biopsy. Alcohol consumption was assessed at baseline and follow up, with heavy episodic drinking assessed in addition to weekly consumption. Primary outcome was significant fibrosis progression, defined as progression by one or more fibrosis stage or the development of end stage liver disease during follow up. Although higher weekly alcohol consumption showed some tendency to predict fibrosis progression (OR for increase in grams of alcohol per week 1.012 (1.000 - 1.025)) only the presence of heavy episodic drinking (defined as >60g/day in men and >48g/day in women more than once a month) reached statistical significance in predicting fibrosis progression.

Of note in both the Ajmera and Ekstedt studies were the very low levels of alcohol consumption in the ‘moderate drinkers’, with the majority (78%) of the moderate drinkers drinking less than monthly in the Ajmera study and the average weekly alcohol consumption in the Ekstedt study being only 39 g/week. Both studies also included a significant number of patients who already had liver inflammation (NASH) at baseline (over 50% in both studies), indicating a higher proportion of patients with a tendency to progressive disease as compared to a general NAFLD population, as would be expected with biopsy-based studies.

In contrast to the relatively selective biopsy studies, Chang et al (59) studied a large prospective population cohort (Kangbuk Samsung Health Study) of whom 58,927 had ultrasound evidence of fatty liver but without evidence of other liver diagnoses or advanced disease. Alcohol exposure was weekly units at baseline and follow up was for a median of 8.3 years with outcome of interest being progression to advanced liver fibrosis using non-invasive blood-based markers of disease. For moderate drinkers (10 - 30 g/day), the risk of progressing to advanced fibrosis (using intermediate/high Fib4 score as the outcome) was HR 1.33 (1.13 - 1.57), when compared to abstainers. Light drinkers (1-10 g/day) showed a tendency towards more advanced disease when compared to abstainers, but this did not reach statistical significance (HR 1.08 CI 0.91-1.27).

Moderate alcohol consumption (below the threshold that would be consistent with ARLD) and risk of liver disease progression in NAFLD

For peer review only - http://bmjopen.bmj.com/site/about/guidelines.xhtml

Three studies extended the definition of moderate alcohol consumption beyond the international consensus definition of ‘moderate consumption’. Two of the studies were rated as having a low risk of bias (57,61), with one rated as high risk of bias (56).

The general population longitudinal data presented by Chang et al (59) is supplemented by two recent related studies by Aberg et al (56,57), using data from the same Finnish National Health Surveys (FINRISK, Health 2000) cohort. The definition of moderate alcohol consumption was increased to include anything up to 50 grams per day in these studies. Although the exposures and outcome measures were the same in the two related studies, the NAFLD population was defined using different fatty liver index (FLI) cut offs values, generating overlapping but distinct study populations. For this reason, data are presented from both studies.
The first study, only available as a conference abstract, (56) used a FLI>30 to retrospectively define their NAFLD population. This low FLI would generally be used as a ‘rule out’ rather than ‘rule in’ cutoff for NAFLD diagnosis (62) and the limited data presented suggests that using abstinence as a reference, any increase in alcohol consumption by 10 g/day, increased incident liver events (combined fatal and non-fatal outcomes) by 43% with a presented HR of 1.43 (1.12 - 1.82) for each 10 g rise in daily alcohol consumption. The data presented contained few details of adjustment factors or analysis plan. This study was graded as having a high risk of bias, and these results should be interpreted with caution.

A larger study (57), based on the same cohort, retrospectively identified a NAFLD population based on a FLI of >60 (the accepted and validated cut off for making a positive diagnosis of NAFLD in the literature (63)). Alcohol intake at cohort entry was based on estimated consumption over the previous year. Lifetime abstainers were used as the reference group. Fatal and non-fatal liver outcomes were studied in 8345 participants over 92 350 person years of follow up. The study concluded that incident liver disease is higher at all levels of alcohol consumption, compared to lifetime abstainers with steadily rising hazard ratios as the level of alcohol consumption increases. Although drinking up to 10g per day was not statistically significantly different to abstaining (HR 1.38 CI 0.74 -2.58 in the final model), levels of alcohol consumption between 10 g and 19 g, which are roughly equivalent to the 14 units per week recommended limits, prognosticated for over double the number of incident liver events in NAFLD patients (HR 2.18 CI 1.05-4.53). At higher levels, which would not necessarily trigger a liver assessment for alcohol related harm in current guidelines, risk of significant liver disease

For peer review only

was nearly nine times higher (for consumption of 40 - 49 g of alcohol a day, HR 8.79 CI 3.95-19.56).

A retrospective Japanese cohort study (61) also looked at stepwise rises in daily alcohol consumption as a prognostic factor for the more specific outcome of hepatocellular carcinoma (HCC) in people with fatty liver (identified on ultrasound). The Kawamura study with 9959 participants followed for a median of nearly 2000 days, had a reference group of people drinking < 20g of alcohol per day, rather than abstainers. This differed from all the other studies reviewed. Only those drinking at between 40-69 g of alcohol a day had a statistically significant increase in rates of HCC (HR 2.48 CI 1.01 - 6.05 p 0.047), with no effect in those drinking at more moderate levels. The population in this retrospective cohort were patients undergoing ultrasound at two tertiary hepatology centres in Japan rather than a general population cohort, and as HCC is known to occur in non-cirrhotic NAFLD (64) comparison with outcomes from other studies should be interpreted with caution.

Excluding the only study rated as having a high risk of bias (56), the other good quality longitudinal studies of varying design, all reported either no association or a negative impact of moderate amounts of alcohol on future liver disease outcomes. This was seen across the studies looking at levels of alcohol consumption within the international definition of moderate consumption, and those that extended this definition of moderate consumption.

Discussion:

Summary of results

In this systematic review of the latest available longitudinal data, we found evidence to suggest that any amount of alcohol, even at low levels, may be harmful for liver health in people with diagnosed NAFLD. This evidence comes from both general population-based cohorts using coded liver outcomes, as well as tertiary centre NAFLD populations defined using histological end points.

Comparison with existing literature

For peer review only

Until recently the majority of evidence in this area has come from cross-sectional studies where alcohol exposure was assessed at the same time as liver outcomes. These data provide somewhat contradictory results, with several studies indicating that moderate alcohol consumption is associated with lower levels of liver disease progression (39,43,65,66) although more recent studies support of our findings, and suggest the opposite (42,45). The design employed in these studies does not allow the assessment of temporal relationships and is open to reverse causality (those with liver damage may be newly abstaining from alcohol for example) in addition to recall and other biases. On the basis of these limitations, cross-sectional studies were excluded from this current review, although they have been widely cited in previous critical reviews in this area, before more recent longitudinal data were available.

In the historical absence of large prospective cohort studies and the impossibility of conducting a controlled trial in the area, comparative work has been undertaken using Mendelian randomisation. This utilises random genetic variations which affects the rate of alcohol metabolism as a proxy measure for alcohol exposure, with randomisation of NAFLD patients based on an allele known to confer lower lifetime alcohol consumption by necessity due to the unpleasant effects of drinking even low levels of alcohol. Findings from this study were supportive of our review, with the group with higher lifetime alcohol consumption showing markers of more severe disease on biopsy, even though alcohol consumption was at very modest levels (46).

In addition to the evidence on the relationship between modest alcohol consumption in NAFLD and liver outcomes, other published studies have focused on overall mortality and cardiovascular outcomes. A study of 4264 participants in an ultrasound diagnosed NAFLD cohort study showed no significant difference in overall mortality in those with alcohol consumption in the low/moderate range versus abstinence after 20 years of follow up (36). A subsequent study with the same US cohort reported a protective effect of low alcohol consumption on overall survival in NAFLD (67). The evidence for a protective effect of low alcohol consumption on cardiovascular outcomes in the general population is generally accepted (68). The evidence for cardiovascular protection in those with NAFLD is more limited, with some evidence that moderate alcohol may provide some benefit (69) but more recent studies finding no protective effects (42,70). The comparative evidence on overall mortality and cardiovascular outcomes highlights the need to assess liver disease risks within these competing contexts.

For peer review only

Strengths and limitations

Although there have been several recent critical reviews of the role of moderate alcohol consumption in NAFLD, the most recent of which reach similar conclusions (4,49,71), these have been wider in their remit with less well-defined inclusion criteria and less systematic methodology. The predetermined inclusion criteria, robust systematic data collection and reporting techniques (in line with PRISMA guidelines) and decision to avoid cross-sectional data are all important in providing the best available evidence to answer the review question of the temporal relationship between moderate alcohol consumption and liver outcomes in NAFLD. The challenges of synthesising observational data, including unmeasured confounding and heterogeneity, were anticipated, but meant that data pooling was not possible.

A particular limitation hindering comparison between studies were the methods of defining moderate alcohol consumption. The consensus for defining a level of alcohol consumption above which a diagnosis of pure NAFLD cannot be made have been supported by the European Association for the Study of the Liver (EASL) and the American Association for the Study of the Liver Diseases (AASLD) and set at 20g/day in women and 30g/day in men (62,72), yet most of the published studies do not use these cut-offs in their data. Until this is standardised across studies, with an additional consensus defining levels above this 'moderate' but not high enough to reach levels associated with a definite diagnosis of ARLD, synthesising the evidence in this area will remain challenging.

Implications for research/practice

This review adds weight to individual studies showing that any level of alcohol intake in NAFLD may be harmful to liver health. Further prospective cohort studies are needed, with detailed definitions/measures of alcohol exposure, and validated clinical liver outcomes, measured at appropriate times. Future research should focus on looking at outcomes in relation to accepted alcohol intake levels used in definitions of NAFLD. It should also take into account that the clinical reality is a dual-aetiology patient who may currently be excluded from both diagnostic categories based on their alcohol intake being too high for NAFLD, and too low for ARLD definitions. This is an ever-expanding patient group seen in many clinical settings.

For peer review only

Based on a synthesis of the evidence presented in this review, clinicians seeing patients with NAFLD in primary or secondary care should currently advise abstinence from alcohol to avoid accelerating liver harm. This is likely to be difficult for patients to accept, and public health messaging will need careful thought if it is to have any impact on liver health.

Author contribution statement:

HJ: conceptualization, methodology, conduct, analysis, writing of initial draft
HO: methodology, conduct, figures/infographic, critical review and comments on drafts
DC: supervision of methodology, conduct, critical review and comments on drafts
DS: methodology, conduct, critical review and comments on drafts
BH: conceptualization, supervision of methodology, conduct, analysis, critical review and comments on drafts
QA: conceptualization, supervision of conduct and analysis, critical review and comments on drafts

Competing interest statement:

All authors have completed the Unified Competing Interest form and declare that the study was funded by an NIHR Doctoral Research Fellowship (HJ personal award). There were no financial relationships with any organisations that might have an interest in the submitted work in the previous three years. All authors report no other relationships or activities that could appear to have influenced the submitted work.

HJ reports grants from National Institute for Health research (NIHR), during the conduct of the study; personal fees from Intercept Pharma, personal fees from Norgine , outside the submitted work;
QMA reports grants from European Commission, during the conduct of the study; other from Acuitas Medical, grants, personal fees and other from Allergan/Tobira, other from E3Bio, other from Eli Lilly & Company Ltd, other from Galmed, grants, personal fees and other from Genfit SA, personal fees and other from Gilead, other from Grunthal, other from Imperial Innovations, grants and other from Intercept Pharma Europe Ltd, other from Inventiva, other from Janssen, personal fees from Kenes, other from MedImmune, other from NewGene, grants and other from Pfizer Ltd, other from Raptor Pharma, grants from GlaxoSmithKline, grants and other from Novartis Pharma AG, grants from Abbvie, personal fees from BMS, grants from GSK, other from NGMBio, other from Madrigal, other from Servier, outside the submitted work;

Funding statement:

This work was supported by and NIHR Doctoral Research Fellowship – HJ personal award. Award no: NIHR300716.

For peer review only

Data Sharing statement:

All data relevant to the study are included in the article or uploaded as supplementary information

Ethics approval statement:

Ethical approval was not required for this study as it involved synthesising existing data and did not involve any human or animal participants.

For peer review only

References:

1. Williams R, Aspinall R, Bellis M, et al. Addressing liver disease in the UK: a blueprint for attaining excellence in health care and reducing premature mortality from lifestyle issues of excess consumption of alcohol, obesity, and viral hepatitis. Lancet. 2014;384(9958):1953–97.
2. GBD 2017 Cirrhosis Collaborators. The global, regional, and national burden of cirrhosis by cause in 195 countries and territories, 1990-2017: a systematic analysis for the Global Burden of Disease Study 2017. Lancet Gastroenterol Hepatol. 2020 Mar;5(3):245-266.
3. Martinez R, Lloyd-Sherlock P, Soliz P, et al. Trends in premature avertable mortality from non-communicable diseases for 195 countries and territories, 1990-2017: a population-based study. Lancet Glob Health 2020;8:e511-e523.
4. Boyle M, Masson S, Anstee QM. The bidirectional impacts of alcohol consumption and the metabolic syndrome: Cofactors for progressive fatty liver disease. J Hepatol. 2018;68(2):251–67.
5. Williams R, Alexander G, Armstrong I, et al. Disease burden and costs from excess alcohol consumption, obesity, and viral hepatitis: fourth report of the Lancet Standing Commission on Liver Disease in the UK. Lancet. 2017 Nov 28;
6. Baumeister SE, Alte D, Meyer C, John U. Health Risk drinking and problematic consumption of alcohol in Pomerania: comparative analysis of the Study of Health in Pomerania (SHIP) compared with the Federal German Health and Examination Survey in 1998. Gesundheitswesen. 2005 Jan;67(1):39–47.

7. Collaborators GBDA. Alcohol use and burden for 195 countries and territories, 1990-2016: a systematic analysis for the Global Burden of Disease Study 2016. Lancet 2018;392:1015-1035.
8. UK Chief Medical Officers' Low Risk Drinking Guidelines. Available at: https://assets.publishing.service.gov.uk/government/uploads/system/uploads/attachment_data/file/545937/UK_CMOs__report.pdf
9. Cirrhosis in over 16s: assessment and management | Guidance and guidelines | NICE. Available from: https://www.nice.org.uk/guidance/ng50
10. Hart CL, Morrison DS, Batty GD, Mitchell RJ, Davey Smith G. Effect of body mass index and alcohol consumption on liver disease: analysis of data from two prospective cohort studies. BMJ. 2010 Mar 11;340:c1240.
11. Liu B, Balkwill A, Reeves G, Beral V, Million Women Study Collaborators. Body mass index and risk of liver cirrhosis in middle aged UK women: prospective study. BMJ. 2010 Mar 11;340:c912.
12. Thun MJ, Peto R, Lopez AD, et al. Alcohol consumption and mortality among middle-aged and elderly U.S. adults. N Engl J Med. 1997 Dec 11;337(24):1705–14.
13. Sookoian S, Castaño GO, Pirola CJ. Modest alcohol consumption decreases the risk of non-alcoholic fatty liver disease: a meta-analysis of 43 175 individuals. Gut. 2014 Mar;63(3):530–2.
14. Tarantino G, Citro V, Capone D. Nonalcoholic Fatty Liver Disease: A Challenge from Mechanisms to Therapy. Journal of Clinical Medicine. 2020 Jan;9(1):15. Available from: https://www.mdpi.com/2077-0383/9/1/15
15. Moher D, Liberati A, Tetzlaff J, Altman DG, The PRISMA Group (2009). *Preferred Reporting Items for Systematic Reviews and Meta-Analyses:* The PRISMA Statement. PLoS Med 6(7): e1000097. doi:10.1371/journal.pmed1000097
16. Ouzzani M, Hammady H, Fedorowicz Z, Elmagarmid A. Rayyan-a web and mobile app for systematic reviews. Syst Rev. 2016 05;5(1):210.
17. Moons KGM, Groot JAH de, Bouwmeester W, et al. Critical Appraisal and Data Extraction for Systematic Reviews of Prediction Modelling Studies: The CHARMS Checklist. PLOS Medicine. 2014 Oct 14;11(10):e1001744.
18. Hayden JA, van der Windt DA, Cartwright JL, Côté P, Bombardier C. Assessing bias in studies of prognostic factors. Ann Intern Med. 2013 Feb 19;158(4):280–6.

19. Popay J, Roberts H, Sowden A, et al. Guidance on the conduct of narrative synthesis in systematic reviews: a product from the ESRC Methods Programme. Lancaster: Lancaster University. 2006;10(2.1):1018–4643
20. Akahane T, Fukui K, Yoshiji H. Light-to-moderate alcohol consumption protects against fatty liver and improves insulin resistance. Hepatology;66:1143A-4A.
21. Akahane T, Namisaki T, Kitade M, Kaji K, Takaya H, Shimozato N, et al. Moderate alcohol consumption protects against fatty liver in males. Journal of Hepatology;68:S841
22. Chang Y, Ryu S, Kim Y, Cho YK, Sung E, Kim HN, et al. Low Levels of Alcohol Consumption, Obesity, and Development of Fatty Liver With and Without Evidence of Advanced Fibrosis. Hepatology;19:19.
23. Colic-Cvrlje V, Naumovski-Mihalic S, Prskalo M, Colic A, Cvjeticanin B, Sabaric B. Prognosis for the patients with alcoholic and nonalcoholic liver disease. Collegium Antropologicum;24:249-52.
24. Cotrim HP, Freitas LA, Alves E, Almeida A, May DS, Caldwell S. Effects of light-to-moderate alcohol consumption on steatosis and steatohepatitis in severely obese patients. European Journal of Gastroenterology & Hepatology 2009;21:969-72.
25. Hara T, Seko Y, Iwai N, Inada Y, Tsuji T, Okuda T, et al. Comparison of the effect of light alcohol consumption on Japanese men with and without fatty liver. Biomedical Reports;11:191-8.
26. Hayashi PH, Harrison SA, Torgerson S, Perez TA, Nochajski T, Russell M. Cognitive lifetime drinking history in nonalcoholic fatty liver disease: some cases may be alcohol related. American Journal of Gastroenterology;99:76-81.
27. Ascha MS, Hanouneh IA, Lopez R, Tamimi TA, Feldstein AF, Zein NN. The incidence and risk factors of hepatocellular carcinoma in patients with nonalcoholic steatohepatitis. Hepatology;51:1972-8.
28. Kimura T, Tanaka N, Fujimori N, Sugiura A, Yamazaki T, Joshita S, et al. Mild drinking habit is a risk factor for hepatocarcinogenesis in non-alcoholic fatty liver disease with advanced fibrosis. World Journal of Gastroenterology;24:1440-50.
29. Moylan CA, Dellinger A, Pang H, Suzuki A, Tillmann HL, Hampton D, et al. Modest alcohol consumption attenuates expression of fibrosis-associated genes in patients with non-alcoholic fatty liver disease (NAFLD). Gastroenterology;1:S914-S5.
30. Patel PJ, Smith D, Connor JP, Horsfall LU, Hayward KL, Hossain F, et al. Alcohol consumption in diabetic patients with nonalcoholic fatty liver disease. Canadian Journal of Gastroenterology and Hepatology 2017;2017 7927685.

For peer review only

31. Patel PJ, Smith DD, Stuart KA, Horsfall LU, Hayward KL, Hossain F, et al. Can modest alcohol intake be dismissed as a co-factor in diabetic patients with non-alcoholic fatty liver disease? Journal of Hepatology 2017;66:S588.
32. Bharmal SJ, Lake J, Boldt M, Watt KD, Jackson S, Hanouneh I. Long-term outcomes of patients with non-obese fatty liver disease (NOFLD): A study from the national health and nutrition examination survey. Gastroenterology;152:S683-S4.
33. Clement P, Ellison RC. Alcohol consumption and risk of death in people with nonalcoholic fatty liver disease. [French]. Revue Medicale Suisse;15:1416
34. Hajifathalian K, McCullough A. Effect of alcohol consumption on survival in nonalcoholic fatty liver disease: A national prospective cohort study. Gastroenterology;152:S1054-S5.
35. Xu L, Xie J, Chen S, Chen Y, Yang H, Miao M, et al. Light-to-Moderate Alcohol Consumption Is Associated With Increased Risk of Type 2 Diabetes in Individuals With Nonalcoholic Fatty Liver Disease: A Nine-Year Cohort Study. The American journal of gastroenterology. 2020;115(6):876-84.
36. Younossi ZM, Stepanova M, Ong J, Yilmaz Y, Duseja A, Eguchi Y, et al. Effects of Alcohol Consumption and Metabolic Syndrome on Mortality in Patients With Nonalcoholic and Alcohol-Related Fatty Liver Disease. Clinical Gastroenterology & Hepatology;17:1625-33.e1
37. Huang A, Chang B, Sun Y, Teng G, Zou ZS. Effect of alcohol consumption on biochemical and histological features of patients with biopsy-proven steatohepatitis. Hepatology;68:1309A
38. Ivanova R, Alexiev A, Mateva L. Fatty liver disease (FLD) and various alcohol intake in patients with obesity. Journal of Gastrointestinal and Liver Diseases 2012;3:20.
39. Mitchell T, Jeffrey GP, MacQuillan GC, Garas G, Ching H, Hamdorf JM, et al. The impact of alcohol consumption on hepatic fibrosis in non-alcoholic fatty liver disease. Hepatology;64:548A.
40. Younossi ZM, Stepanova M, Ong J, Yilmaz Y, Duseja AK, Eguchi Y, et al. Non-alcoholic fatty liver disease (NAFLD) and alcoholic fatty liver disease (AFLD): The impact of alcohol consumption and metabolic syndrome on mortality. Hepatology;68:801A-2A
41. Hagstrom H, Nasr P, Ekstedt M, Kechagias S, Onnerhag K, Nilsson E, et al. Low to moderate lifetime alcohol consumption is associated with less advanced stages of fibrosis in non-alcoholic fatty liver disease. Hepatology;64:18A-9A

42. Kashiwagi K, Yamaguchi A, Shiba S, Taniki N, Inoue N, Takaishi H, et al. Moderate alcohol consumption is not associated with subclinical cardiovascular damage but with hepatic fibrosis in non-alcoholic fatty liver disease. Alcohol. 2020;89:1-7.
43. Kwon HK, Greenson JK, Conjeevaram HS. Effect of lifetime alcohol consumption on the histological severity of non-alcoholic fatty liver disease. Liver International;34:129-35.
44. Mitchell T, Jeffrey GP, de Boer B, MacQuillan G, Garas G, Ching H, et al. Type and Pattern of Alcohol Consumption is Associated With Liver Fibrosis in Patients With Non-alcoholic Fatty Liver Disease. American Journal of Gastroenterology;113:1484-93.
45. Mulazzani L, Alvisi M, Tovoli F, Leoni S, Goio E, Benevento F, et al. Moderate alcohol consumption is associated with risk of fibrosis in patients with non-alcoholic fatty liver disease. Digestive and Liver Disease. 2020;52:e43-e4.
46. Sookoian S, Flichman D, Castano GO, Pirola CJ. Mendelian randomisation suggests no beneficial effect of moderate alcohol consumption on the severity of nonalcoholic fatty liver disease. Alimentary Pharmacology & Therapeutics;44:1224-34
47. Yamada K, Mizukoshi E, Seike T, Horii R, Kitahara M, Sunagozaka H, et al. Light alcohol consumption has the potential to suppress hepatocellular injury and liver fibrosis in non-alcoholic fatty liver disease. PLoS ONE.2018;13:e0191026.
48. Aberg F, Farkkila M. Drinking and Obesity: Alcoholic Liver Disease/Nonalcoholic Fatty Liver Disease Interactions. Seminars in liver disease. 2020;40(2):154-62.
49. Aberg F, Farkkila M, Mannisto V. Interaction Between Alcohol Use and Metabolic Risk Factors for Liver Disease: A Critical Review of Epidemiological Studies. Alcoholism-Clinical and Experimental Research. 2020;44(2):384-403.
50. Aberg F, Puukka P, Salomaa V, Mannisto S, Lundqvist A, Valsta L, et al. Combined Effects of Alcohol and Metabolic Disorders in Patients With Chronic Liver Disease. Clinical Gastroenterology and Hepatology. 2020;18(4):995.
51. Armstrong MJ, Mellinger JL, Trivedi PJ. Alcohol Consumption in Patients with Non-alcoholic Fatty Liver Disease: Convenient vs. Inconvenient Truths. American Journal of Gastroenterology;113:1437-9.
52. Glyn-Owen K, Bohning D, Parkes J, Roderick P, Buchanan R. The combined effect of alcohol and obesity on risk of liver disease: A systematic review and metaanalysis. Hepatology v70 suppl1 2019. 2019;70:753A.
53. Idalsoaga F, Kulkarni AV, Mousa OY, Arrese M, Arab JP. Non-alcoholic Fatty Liver Disease and Alcohol-Related Liver Disease: Two Intertwined Entities. Frontiers in Medicine. 2020;7:448.

54. Petroni ML, Brodosi L, Marchignoli F, Musio A, ra, Marchesini G. Moderate Alcohol Intake in Non-Alcoholic Fatty Liver Disease: To Drink or Not to Drink? Nutrients 2019;11:3048.
55. Weng G, Dunn W. Effect of alcohol consumption on nonalcoholic fatty liver disease. Translational Gastroenterology & Hepatology 2019;4:70.
56. Åberg F, Puukka P, Sahlman P et al. In NAFLD, alcohol drinking habits and genetics predict progression to advanced liver disease: follow-up of population surveys. Journal of Hepatology. 2019; 70: e141. (poster abstract)
57. Åberg F, Puukka P, Salomaa V, et al. Risks of Light and Moderate Alcohol Use in Fatty Liver Disease: Follow-Up of Population Cohorts. Hepatology. 2020 Mar;71(3):835–48.
58. Ajmera V, Belt P, Wilson LA, et al. Among Patients With Nonalcoholic Fatty Liver Disease, Modest Alcohol Use Is Associated With Less Improvement in Histologic Steatosis and Steatohepatitis. Clin Gastroenterol Hepatol. 2018;16(9):1511-1520.e5.
59. Chang Y, Cho YK, Kim Y, et al. Nonheavy Drinking and Worsening of Noninvasive Fibrosis Markers in Nonalcoholic Fatty Liver Disease: A Cohort Study. Hepatology. 2019;69(1):64–75.
60. Ekstedt M, Franzén LE, Holmqvist M, et al. Alcohol consumption is associated with progression of hepatic fibrosis in non-alcoholic fatty liver disease. Scand J Gastroenterol. 2009;44(3):366–74.
61. Kawamura Y, Arase Y, Ikeda K, et al. Effects of Alcohol Consumption on Hepatocarcinogenesis in Japanese Patients With Fatty Liver Disease. Clin Gastroenterol Hepatol. 2016 Apr;14(4):597–605.
62. European Association for the Study of the Liver (EASL), European Association for the Study of Diabetes (EASD), European Association for the Study of Obesity (EASO). EASL-EASD-EASO Clinical Practice Guidelines for the Management of Non-Alcoholic Fatty Liver Disease. Obes Facts. 2016;9(2):65–90.
63. Bedogni G, Bellentani S, Miglioli L, et al. The Fatty Liver Index: a simple and accurate predictor of hepatic steatosis in the general population. BMC Gastroenterol. 2006 Nov 2;6:33.
64. Negro F. Natural history of NASH and HCC. Liver Int. 2020;40 Suppl 1:72–6.
65. Dunn W, Sanyal AJ, Brunt EM, et al. Modest alcohol consumption is associated with decreased prevalence of steatohepatitis in patients with non-alcoholic fatty liver disease (NAFLD). J Hepatol. 2012 Aug;57(2):384–91.

For peer review only

66. Hagström H, Nasr P, Ekstedt M, et al. Low to moderate lifetime alcohol consumption is associated with less advanced stages of fibrosis in non-alcoholic fatty liver disease. Scand J Gastroenterol. 2017 Feb;52(2):159–65.
67. Hajifathalian K, Torabi Sagvand B, McCullough AJ. Effect of Alcohol Consumption on Survival in Nonalcoholic Fatty Liver Disease: A National Prospective Cohort Study. Hepatology. 2019;70(2):511–21.
68. Klatsky AL. Alcohol and cardiovascular diseases. Expert Review of Cardiovascular Therapy [Internet]. 2009 May 1 [cited 2020 Sep 14];7(5):499–506.
69. Sinn DH, Gwak G-Y, Cho J, et al. Modest alcohol consumption and carotid plaques or carotid artery stenosis in men with non-alcoholic fatty liver disease. Atherosclerosis. 2014 Jun 1;234(2):270–5.
70. VanWagner LB, Ning H, Allen NB, et al. Alcohol Use and Cardiovascular Disease Risk in Patients With Nonalcoholic Fatty Liver Disease. Gastroenterology. 2017;153(5):1260-1272.e3.
71. Ajmera VH, Terrault NA, Harrison SA. Is moderate alcohol use in nonalcoholic fatty liver disease good or bad? A critical review. Hepatology. 2017 Jun;65(6):2090–9.
72. Chalasani N, Younossi Z, Lavine JE, et al. The diagnosis and management of non-alcoholic fatty liver disease: practice guideline by the American Gastroenterological Association, American Association for the Study of Liver Diseases, and American College of Gastroenterology. Gastroenterology. 2012 Jun;142(7):1592–609.

Figure 1: PRISMA diagram of study selection

For peer review only

Fig 1: PRISMA diagram of study selection
7

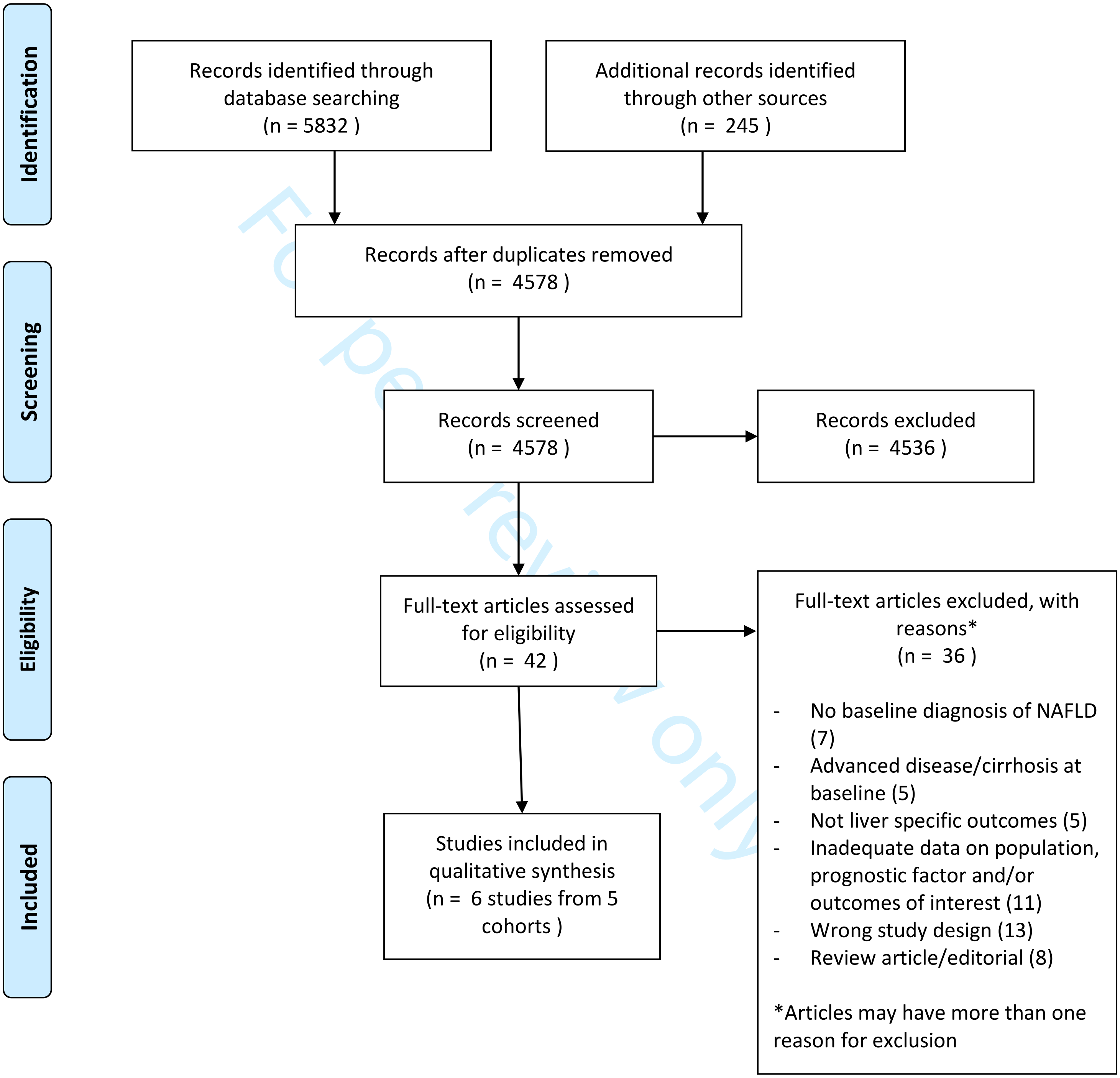

For peer review only

For peer review only - http://bmjopen.bmj.com/site/about/guidelines.xhtml

Medline(Ovid)

1. (((fatty or fat or steato*) adj3 (liver* or hepat*)) or steatohepat* or (visceral adj2 steato*)).ti,ab.
2. non-alcoholic fatty liver disease/
3. fatty liver/
4. (nafl* or nash).ti,ab.
5. non?alcoholic steato*.mp. [mp=title, abstract, original title, name of substance word, subject heading word, floating sub-heading word, keyword heading word, organism supplementary concept word, protocol supplementary concept word, rare disease supplementary concept word, unique identifier, synonyms]
6. (non?alcoholic adj3 (liver or fat*)).mp. [mp=title, abstract, original title, name of substance word, subject heading word, floating sub-heading word, keyword heading word, organism supplementary concept word, protocol supplementary concept word, rare disease supplementary concept word, unique identifier, synonyms]
7. or/1-6
8. exp Alcohol Drinking/
9. Alcoholism/ or Alcoholic Beverages/
10. (alcohol adj2 (unit* or consum* or level* or mg or g)).ti,ab.
11. (moderat* adj3 alcohol*).mp. [mp=title, abstract, original title, name of substance word, subject heading word, floating sub-heading word, keyword heading word, organism supplementary concept word, protocol supplementary concept word, rare disease supplementary concept word, unique identifier, synonyms]
12. or/8-11
13. 7 and 12
14. liver disease.mp. [mp=title, abstract, original title, name of substance word, subject heading word, floating sub-heading word, keyword heading word, organism supplementary concept word, protocol supplementary concept word, rare disease supplementary concept word, unique identifier, synonyms]
15. fibrosis.mp. [mp=title, abstract, original title, name of substance word, subject heading word, floating sub-heading word, keyword heading word, organism supplementary concept word, protocol supplementary concept word, rare disease supplementary concept word, unique identifier, synonyms]
16. (scar* adj3 liver).mp. [mp=title, abstract, original title, name of substance word, subject heading word, floating sub-heading word, keyword heading word, organism supplementary concept word, protocol supplementary concept word, rare disease supplementary concept word, unique identifier, synonyms]
17. cicatrix.mp. [mp=title, abstract, original title, name of substance word, subject heading word, floating sub-heading word, keyword heading word, organism supplementary concept word, protocol supplementary concept word, rare disease supplementary concept word, unique identifier, synonyms]
18. (end-stage adj3 liver).mp. [mp=title, abstract, original title, name of substance word, subject heading word, floating sub-heading word, keyword heading word, organism supplementary concept word, protocol supplementary concept word, rare disease supplementary concept word, unique identifier, synonyms]
19. ((liver or biliary) adj cirrhosis).mp. [mp=title, abstract, original title, name of substance word, subject heading word, floating sub-heading word, keyword heading word, organism supplementary concept word, protocol supplementary concept word, rare disease supplementary concept word, unique identifier, synonyms]
20. (hepatic insufficiency or liver failure or end stage liver disease or hepatic failure or hepatic encephalopathy or hepatic impairment).mp. [mp=title, abstract, original title, name of substance

word, subject heading word, floating sub-heading word, keyword heading word, organism supplementary concept word, protocol supplementary concept word, rare disease supplementary concept word, unique identifier, synonyms]
21. (mortality or death or dead or deceased or passed away).mp. [mp=title, abstract, original title, name of substance word, subject heading word, floating sub-heading word, keyword heading word, organism supplementary concept word, protocol supplementary concept word, rare disease supplementary concept word, unique identifier, synonyms]
22. ((hepatocellular or liver cell) adj carcinoma).mp. [mp=title, abstract, original title, name of substance word, subject heading word, floating sub-heading word, keyword heading word, organism supplementary concept word, protocol supplementary concept word, rare disease supplementary concept word, unique identifier, synonyms]
23. or/14-22
24. 13 and 23
25. limit 24 to yr="1990-Current"

Embase(Ovid)

1. (((fatty or fat or steato*) adj3 (liver* or hepat*)) or steatohepat* or (visceral adj2 steato*)).ti,ab.
2. exp nonalcoholic fatty liver/
3. (nafl* or nash).ti,ab.
4. non?alcoholic steato*.mp. [mp=title, abstract, heading word, drug trade name, original title, device manufacturer, drug manufacturer, device trade name, keyword, floating subheading word, candidate term word]
5. (non?alcoholic adj3 (liver or fat*)).mp. [mp=title, abstract, heading word, drug trade name, original title, device manufacturer, drug manufacturer, device trade name, keyword, floating subheading word, candidate term word]
6. or/1-5
7. drinking behavior/
8. exp alcoholism/
9. exp alcoholic beverage/
10. (alcohol adj2 (unit* or consum* or level* or mg or g)).ti,ab.
11. (moderat* adj3 alcohol*).mp. [mp=title, abstract, heading word, drug trade name, original title, device manufacturer, drug manufacturer, device trade name, keyword, floating subheading word, candidate term word]
12. or/7-11
13. 6 and 12
14. liver disease.mp. [mp=title, abstract, heading word, drug trade name, original title, device manufacturer, drug manufacturer, device trade name, keyword, floating subheading word, candidate term word]
15. fibrosis.mp. [mp=title, abstract, heading word, drug trade name, original title, device manufacturer, drug manufacturer, device trade name, keyword, floating subheading word, candidate term word]
16. (scar* adj3 liver).mp. [mp=title, abstract, heading word, drug trade name, original title, device manufacturer, drug manufacturer, device trade name, keyword, floating subheading word, candidate term word]
17. cicatrix.mp. [mp=title, abstract, heading word, drug trade name, original title, device manufacturer, drug manufacturer, device trade name, keyword, floating subheading word, candidate term word]

18. ((hepatcellular or liver cell) adj carcinoma).mp. [mp=title, abstract, heading word, drug trade name, original title, device manufacturer, drug manufacturer, device trade name, keyword, floating subheading word, candidate term word]
19. (end-stage adj3 liver).mp. [mp=title, abstract, heading word, drug trade name, original title, device manufacturer, drug manufacturer, device trade name, keyword, floating subheading word, candidate term word]
20. (Mortality or death or dead or deceased or passed away).mp. [mp=title, abstract, heading word, drug trade name, original title, device manufacturer, drug manufacturer, device trade name, keyword, floating subheading word, candidate term word]
21. ((liver or biliary) adj cirrhosis).mp. [mp=title, abstract, heading word, drug trade name, original title, device manufacturer, drug manufacturer, device trade name, keyword, floating subheading word, candidate term word]
22. (hepatic insufficiency or liver failure or end stage liver disease or hepatic failure or hepatic encephalopathy or hepatic impairment).mp. [mp=title, abstract, heading word, drug trade name, original title, device manufacturer, drug manufacturer, device trade name, keyword, floating subheading word, candidate term word]
23. or/14-22
24. 13 and 23
25.
26. limit 25 to yr="1990 -Current"

Cochrane library

ID Search Hits
#1 (((fatty or fat or steato*) NEAR/3 (liver* or hepat*)) or steatohepat* or (visceral NEAR/2 steato*))
#2 MeSH descriptor: [Non-alcoholic Fatty Liver Disease] this term only
#3 MeSH descriptor: [Fatty Liver] this term only
#4 (nafl* or nash)
#5 non?alcoholic steato*
#6 (non?alcoholic NEAR/3 (liver or fat*))
#7 #1 or #2 or #3 or #4 or #5 or #6
#8 MeSH descriptor: [Alcohol Drinking] explode all trees
#9 MeSH descriptor: [Alcoholism] this term only
#10 MeSH descriptor: [Alcoholic Beverages] this term only
#11 (alcohol NEAR/2 (unit* or consum* or level* or mg or g))
#12 (moderat* NEAR/3 alcohol)
#13 #8 or #9 or #10 or #11 or #12
#14 #7 AND #13
#15 Liver disease
#16 Fibrosis
#17 cicatrix or (scar* NEAR/3 liver)
#18 (end-stage NEAR/3 liver)
#19 ((Liver or biliary) NEAR cirrhosis)
#20 (hepatic insufficiency or liver failure or end stage liver disease or hepatic failure or hepatic encephalopathy or hepatic impairment)
#21 (Mortality or death or dead or deceased or passed away)
#22 ((hepatocellular or liver cell) NEAR carcinoma)
#23 #15 or #16 or #17 or #18 or #19 or #20 or #21 or #22

For peer review only - http://bmjopen.bmj.com/site/about/guidelines.xhtml

#24 #14 AND #23 with Cochrane Library publication date Between Jan 1990 and Dec 2019

CINAHL(EBSCO)

S1 TI ((((fatty or fat or steato*) N3 (liver* or hepat*)) or steatohepat* or (visceral N2 steato*))) OR AB ((((fatty or fat or steato*) N3 (liver* or hepat*)) or steatohepat* or (visceral N2 steato*))) OR (MH "Nonalcoholic Fatty Liver Disease") OR (MH "Fatty Liver") OR TI ((nafl* or nash)) OR AB ((nafl* or nash)) OR non#alcoholic steato* OR ((non-alcoholic N3 (liver or fat*)))
S2 (MH "Alcoholic Drinking+") OR (((MH "Alcoholism") or (MH "Alcoholic Beverages"))) OR TI ((alcohol N2 (unit* or consum* or level* or mg or g))) OR AB ((alcohol N2 (unit* or consum* or level* or mg or g))) OR AB (moderat* N3 alcohol)
S3 S1 AND S2
S4 (Liver Diseases OR Liver Failure OR ((Liver or biliary) N1 Cirrhosis) OR Fibrosis OR Cicatrix OR (scar* N3 liver) OR (end-stage N3 liver) OR hepatic insufficiency OR end stage liver disease OR hepatic failure OR hepatic encephalopathy OR hepatic impairment OR Mortality OR death OR dead OR deceased OR passed away OR ((Hepatocellular OR liver cell) N1 carcinoma))
S5 S3 AND S4

Web of Science (CPCI)

1. TS = (((fatty or fat or steato*_ NEAR/3 (liver* or hepat*)) or steatohepat* or (visceral NEAR/2 steato*))
2. TS = non-alcoholic fatty liver disease
3. TS = fatty liver
4. TS = (nafl* or nash)
5. TS = non-alochol steato*
6. TS = (non-alcoholic NEAR/3 (liver or fat*))
7. #1 OR #2 OR #3 OR #4 OR #5 OR #6
8. TS = alcohol drinking
9. TS = (alcoholism or alcoholic beverages)
10. TS = (alcohol NEAR/2 (unit* or consum* or level* or mg or g))
11. TS = (moderate NEAR/3 alcohol*)
12. #8 OR #9 OR #10 OR #11
13. #7 AND #12
14. TS = liver disease
15. TS = fibrosis
16. TS = cicatrix
17. TS = (end-stage NEAR/3 liver)
18. TS = liver cirrhosis
19. TS = hepatic insufficiency
20. #14 OR #15 OR #16 OR #17 OR #18 OR #19
21. #13 AND #20

PRISMA 2020 Checklist

Section and Topic	Item #	Checklist item	Location where item is reported
**TITLE**			
Title	1	Identify the report as a systematic review.	title
**ABSTRACT**			
Abstract	2	See the PRISMA 2020 for Abstracts checklist.	P 2-3
**INTRODUCTION**			
Rationale	3	Describe the rationale for the review in the context of existing knowledge.	P3-5
Objectives	4	Provide an explicit statement of the objective(s) or question(s) the review addresses.	P 5(end)
**METHODS**			
Eligibility criteria	5	Specify the inclusion and exclusion criteria for the review and how studies were grouped for the syntheses.	P 6 (para 1-2)
Information sources	6	Specify all databases, registers, websites, organisations, reference lists and other sources searched or consulted to identify studies. Specify the date when each source was last searched or consulted.	P 6 -7
Search strategy	7	Present the full search strategies for all databases, registers and websites, including any filters and limits used.	supplementary data
Selection process	8	Specify the methods used to decide whether a study met the inclusion criteria of the review, including how many reviewers screened each record and each report retrieved, whether they worked independently, and if applicable, details of automation tools used in the process.	end P6 - P7
Data collection process	9	Specify the methods used to collect data from reports, including how many reviewers collected data from each report, whether they worked independently, any processes for obtaining or confirming data from study investigators, and if applicable, details of automation tools used in the process.	end P6-7
Data items	10a	List and define all outcomes for which data were sought. Specify whether all results that were compatible with each outcome domain in each study were sought (e.g. for all measures, time points, analyses), and if not, the methods used to decide which results to collect.	P7
	10b	List and define all other variables for which data were sought (e.g. participant and intervention characteristics, funding sources). Describe any assumptions made about any missing or unclear information.	P7
Study risk of bias assessment	11	Specify the methods used to assess risk of bias in the included studies, including details of the tool(s) used, how many reviewers assessed each study and whether they worked independently, and if applicable, details of automation tools used in the process.	P7
Effect measures	12	Specify for each outcome the effect measure(s) (e.g. risk ratio, mean difference) used in the synthesis or presentation of results.	table 2
Synthesis methods	13a	Describe the processes used to decide which studies were eligible for each synthesis (e.g. tabulating the study intervention characteristics and comparing against the planned groups for each synthesis (item #5)).	P7
	13b	Describe any methods required to prepare the data for presentation or synthesis, such as handling of missing summary statistics, or data conversions.	N/A
	13c	Describe any methods used to tabulate or visually display results of individual studies and syntheses.	table 2
	13d	Describe any methods used to synthesize results and provide a rationale for the choice(s). If meta-analysis was performed, describe the model(s), method(s) to identify the presence and extent of statistical heterogeneity, and software package(s) used.	narrative
	13e	Describe any methods used to explore possible causes of heterogeneity among study results (e.g. subgroup analysis, meta-regression).	N/A
	13f	Describe any sensitivity analyses conducted to assess robustness of the synthesized results.	N/A

For peer review only

For peer review only - http://bmjopen.bmj.com/site/about/guidelines.xhtml

PRISMA 2020 Checklist

Reporting bias assessment	14	Describe any methods used to assess risk of bias due to missing results in a synthesis (arising from reporting biases).	ROB QUIPS used - P7
Certainty assessment	15	Describe any methods used to assess certainty (or confidence) in the body of evidence for an outcome.	ROB - QUIPS used - P7
**RESULTS**			
Study selection	16a	Describe the results of the search and selection process, from the number of records identified in the search to the number of studies included in the review, ideally using a flow diagram.	Fig 1 and para 1 of results P7-8
	16b	Cite studies that might appear to meet the inclusion criteria, but which were excluded, and explain why they were excluded.	beginning 8 and box in PRISMA diagram (fig 1)
Study characteristics	17	Cite each included study and present its characteristics.	Table 2
Risk of bias in studies	18	Present assessments of risk of bias for each included study.	Table 2
Results of individual studies	19	For all outcomes, present, for each study: (a) summary statistics for each group (where appropriate) and (b) an effect estimate and its precision (e.g. confidence/credible interval), ideally using structured tables or plots.	Table 2
Results of syntheses	20a	For each synthesis, briefly summarise the characteristics and risk of bias among contributing studies.	Table 2 and results narrative P10-14
	20b	Present results of all statistical syntheses conducted. If meta-analysis was done, present for each the summary estimate and its precision (e.g. confidence/credible interval) and measures of statistical heterogeneity. If comparing groups, describe the direction of the effect.	N/A
	20c	Present results of all investigations of possible causes of heterogeneity among study results.	N/A
	20d	Present results of all sensitivity analyses conducted to assess the robustness of the synthesized results.	N/A
Reporting biases	21	Present assessments of risk of bias due to missing results (arising from reporting biases) for each synthesis assessed.	N/A
Certainty of evidence	22	Present assessments of certainty (or confidence) in the body of evidence for each outcome assessed.	N/A
**DISCUSSION**			
Discussion	23a	Provide a general interpretation of the results in the context of other evidence.	p16 - 1st paragraph discussion
	23b	Discuss any limitations of the evidence included in the review.	P17
	23c	Discuss any limitations of the review processes used.	P17
	23d	Discuss implications of the results for practice, policy, and future research.	P18
**OTHER INFORMATION**			
Registration and	24a	Provide registration information for the review, including register name and registration number, or state that the review was not registered.	abstract and 1st line

For peer review only

PRISMA 2020 Checklist

protocol			methods (P5/6)
	24b	Indicate where the review protocol can be accessed, or state that a protocol was not prepared.	methods P5/6
	24c	Describe and explain any amendments to information provided at registration or in the protocol.	N/A
Support	25	Describe sources of financial or non-financial support for the review, and the role of the funders or sponsors in the review.	abstract and end of text (p19)
Competing interests	26	Declare any competing interests of review authors.	p18-19
Availability of data, code and other materials	27	Report which of the following are publicly available and where they can be found: template data collection forms; data extracted from included studies; data used for all analyses; analytic code; any other materials used in the review.	search strat available - supplement ary material.

From: Page MJ, McKenzie JE, Bossuyt PM, Boutron I, Hoffmann TC, Mulrow CD, et al. The PRISMA 2020 statement: an updated guideline for reporting systematic reviews. BMJ 2021;372:n71. doi: 10.1136/bmj.n71

For more information, visit: http://www.prisma-statement.org/

For peer review only
